# The Feasibility and User-Experience of a Digital Health Intervention Designed to Prevent Weight Gain in New Kidney Transplant Recipients—The ExeRTiOn2 Trial

**DOI:** 10.3389/fnut.2022.887580

**Published:** 2022-05-23

**Authors:** Ellen M. Castle, Giulia Dijk, Elham Asgari, Sapna Shah, Rachel Phillips, James Greenwood, Kate Bramham, Joseph Chilcot, Sharlene A. Greenwood

**Affiliations:** ^1^Therapies Department, King's College Hospital, NHS Foundation Trust, London, United Kingdom; ^2^King's Kidney Care, King's College Hospital, London, United Kingdom; ^3^Renal Sciences, King's College London University, London, United Kingdom; ^4^Department of Nutrition and Dietetics, Hammersmith Hospital, Imperial College Healthcare NHS Trust, London, United Kingdom; ^5^Kidney Services Team, Guy's and St Thomas' NHS Foundation Trust London, London, United Kingdom; ^6^Imperial Clinical Trials Unit, School of Public Health, Imperial College London, London, United Kingdom; ^7^Pragmatic Clinical Trials Unit, Centre for Evaluation and Methods, Wolfson Institute of Population Health, London, United Kingdom; ^8^Victor Horsley Department of Neurosurgery, University College London Hospital, London, United Kingdom; ^9^Department of Women and Children's Health, Faculty of Life Sciences and Medicine King's College London, London, United Kingdom; ^10^Department of Psychology, Institute of Psychiatry, Psychology & Neuroscience, King's College London, London, United Kingdom

**Keywords:** web-based intervention, weight gain prevention, physical activity, kidney transplantation, behavior change

## Abstract

Half of kidney transplant recipients (KTRs) gain more than 5% of their body weight in the first year following transplantation. KTRs have requested support with physical activity (PA) and weight gain prevention, but there is no routine care offered. There are few high-quality studies investigating the clinical value of diet, PA or combined interventions to prevent weight gain. The development and evaluation of theoretically informed complex-interventions to mitigate weight gain are warranted. The aims of this mixed-methods randomized controlled trial (RCT) were to explore the feasibility, acceptability and user-experience of a digital healthcare intervention (DHI) designed to prevent post-transplant weight gain, in preparation for a large multi-center trial. New KTRs (<3 months) with access to an internet compatible device were recruited from a London transplant center. The usual care (UC) group received standard dietary and PA advice. The intervention group (IG) received access to a 12-week DHI designed to prevent post-transplant weight gain. Primary feasibility outcomes included screening, recruitment, retention, adherence, safety and hospitalizations and engagement and experience with the DHI. Secondary outcomes (anthropometrics, bioimpedance, arterial stiffness, 6-minute walk distance and questionnaires) were measured at baseline, 3- and 12-months. 38 KTRs were screened, of which 32 (84.2%) were eligible, and of those 20 (62.5%) consented, with 17 participants (85%) completing baseline assessment (Median 49 years, 58.8% male, Median 62 days post-transplant). Participants were randomized using a computer-generated list (*n* = 9 IG, *n* = 8 UC). Retention at 12-months was 13 (76.4%) (*n* = 6 IG, *n* = 7 UC). All *a priori* progression criteria were achieved. There were no associated adverse events. Reflexive thematic analysis revealed four themes regarding trial participation and experience whilst using the DHI. Halting recruitment due to COVID-19 resulted in the recruitment of 40% of the target sample size. Mixed-methods data provided important insights for future trial design. A definitive RCT is warranted and welcomed by KTRs.

**Clinical Trial Registration:**

www.clinicalTrials.gov, identifier: NCT03996551.

## Introduction

Weight gain within the first year of receiving a kidney transplant is a critical health issue (1) and occurs in both obese and non-obese kidney transplant recipients (KTRs) (2). Over half of KTRs gain more than 5% of their body weight within the first year of transplantation (3, 4). Post-transplant weight gain is usually accompanied with an increase fat mass, not lean tissue mass (3). There is a positive association with an increase in adipose tissue (visceral and sub-cutaneous) with insulin resistance in KTRs (5). Factors underlying post kidney transplant weight gain include reduced physical function (6) and physical activity (PA) (7), increased appetite, (3) steroid medication use (8), and the lifting of dietary restrictions (9). Whilst “triple therapy” anti-rejection regimes including steroid medication are current practice to reduce the risk of graft failure (10–12), they have been found to increase both the severity and incidence of cardiovascular risk factors (13). In addition, these medications effect bone health, weight gain, hypertension, abnormal glucose mechanism and the development of post-transplant diabetes mellitus (10, 11). They have also been associated with appetite stimulation and changes in nutrient partitioning that favor fat deposition (14). Therefore, interventions to address weight gain and modifiable risk factors such as physical activity and healthy eating behaviors are warranted (15).

KTRs have asked for support for both PA and healthy eating behaviors post transplantation (9, 16, 17). Despite national clinical practice (18) and workforce practice guidelines (19) that recommend access to both kidney physiotherapists and dietitians, these healthcare professionals (HCPs) are not routinely represented in all transplant centers (20). Whilst COVID-19 has seen an increase in virtual kidney services (21), and the creation of online PA and well-being interventions for people living with chronic kidney disease (22), there remains no recognized intervention to prevent weight gain in new KTRs (15).

A recent systematic review and meta-analyses (15) revealed that there was no evidence that dietary, exercise, or combined interventions led to significant changes in body weight or body mass index (BMI) in a pooled sample of participants within the first year of receiving a kidney transplant. Limitations of the review include small number of randomized-controlled trials (RCTs) with significant methodological variation, and variable quality study design (17). Future studies would benefit from healthcare digital behavior change intervention guidance (23) such as the use of the behavior change techniques (24, 25) and behavior change wheel (26, 27) to explore and report intervention components, and context. There is therefore a need for quality, theory informed RCTs to investigate complex interventions that include dietary counseling, PA interventions and behavior change techniques to address the multifactorial problem of weight gain post kidney transplantation.

The usability and experience of a personalized digital health intervention (DHI): ExeRTiOn (Exercise and weight gain prevention in renal transplant online), which was co-designed with KTRs and transplant healthcare professionals (HCPs) to aid weight gain prevention after kidney transplantation has already been reported (16). The results from this initial study (16) were used to facilitate iterative patient-led refinements and improve the acceptability of the ExeRTiOn DHI in preparation for its use in this feasibility RCT. The aims of this current mixed-methods RCT were to explore the feasibility, acceptability and experience of using the ExeRTiOn DHI, and participating in the trial in preparation for a large multi-center trial.

## Methods

### Trial Design

Mixed-methods feasibility RCT with 1:1 allocation ratio.

### Participants

KTRs were approached during routine transplant clinics at both King's College Hospital NHS Foundation Trust and Guy's and ST Thomas' Hospital NHS Foundation Trust. Participants were included if they were ≥18 years, had received a single organ kidney transplant within <3-months, had access to an internet compatible device, and had a BMI ≥ 18.5 kg/m^2^. Exclusion criteria included active pregnancy, a medical condition preventing PA participation (e.g., unstable angina), a cognitive impairment preventing engagement with the DHI, or if they were unable to complete the DHI in English.

### Study Procedures

Ethical approval was sought, and obtained, from the London Dulwich Research Ethics Committee (19/LO/1138). The trial was registered (www.clinicalTrials.gov; no: NCT03996551). Eligible KTRs were provided with approved patient information sheets and given ≥24 h (or at the participants convenience) to consider participation. Participants provided written consent, attended a baseline assessment, and were then randomized with a computer-generated list (28). They were allocated to either the 12-week ExeRTiOn intervention group (IG) or usual care (UC) by a member of the research team. The trial physiotherapist and participants were not blinded.

Participants attended the King's National Institute for Health Research Clinical Research Facility for assessment of secondary outcomes at baseline, 3-, and 12-months. Medical history and hospital admissions were reviewed. Assessments were booked around clinical appointments with a window of 14 (±7) days. A purposive sample of participants from both groups were invited to complete individual semi-structured interviews, conducted over the telephone or face-to-face.

### Interventions

#### The ExeRTiOn DHI

Participants in the IG were provided with access to the ExeRTiOn DHI. The ExeRTiOn DHI has been previously reported (16). The design, development and evaluation of the ExeRTiOn DHI was iterative, and was informed by the Medical Research Council Framework for complex interventions (29), the combined intervention design approach (30), the person-based approach (31), evidence and theory, guidance for digital healthcare development (23, 32), the behavior change wheel (26), recognized behavior change techniques (24, 25), principles of self-efficacy (33), motivational interviewing (34), patient and public involvement, and input from research and clinical experts such as the renal-specific weight management team (35–37).

In summary the ExeRTiOn DHI was password protected, had both a patient-facing and back-end website monitored by a trial physiotherapist, with a secure encrypted two-way message function between participants and the trial physiotherapist. IG participants were provided with a brief one-to-one orientation session with the trial physiotherapist and were then able to complete the 12-weekly sessions independently with any internet compatible device. As the ExeRTiOn DHI was designed utilizing a reactive website, participants could choose to view the DHI with their smart phone, laptop, tablet or computer. DHI content and functionality included kidney transplant specific education from health care professionals, tips from kidney transplant recipients, an optional home exercise diary, a resource page, graphical displays of self-reported physical activity minutes and body weight, and the secure two-way message function (16). Intervention participants were encouraged to set physical activity and healthy eating goals, and were prompted to self-monitor physical activity minutes and body weight weekly (16). Food intake was not captured.

Personalized “trigger messages” were sent by the trial physiotherapist to the IG participants when two sessions in a row were not completed. Automated reminder emails, and personalized messages were provided as per the research protocol. The physiotherapist who supported the DHI engagement was trained in motivational interviewing principles (34, 38, 39), and had experience working in both weight management and exercise services for people living with a kidney transplant. After completion of the 12-week DHI, IG participants were able to revisit completed sessions until the 12-month visit.

#### Usual Care

Usual care at both sites involved routine inpatient physiotherapy input, the provision of a “healthy eating after kidney transplant” leaflet by a renal dietician during transplant surgery admission, and encouragement to be physically active, and follow a healthy diet from outpatient transplant nephrologists and nurses.

### Primary Feasibility Outcomes

Primary feasibility outcomes included screening, recruitment, retention, adherence to study visits, safety and hospitalizations, engagement and experience whilst using the DHI, and the feasibility and experiences taking part in the study. This would allow the assessment of the feasibility of the DHI but also the feasibility of running a RCT in preparation for a definitive RCT. Feasibility was assessed by a set of *a priori* progression criteria. “Stop” and “go” criteria (40) were decided prior to the intervention by the study team, Trial Management Group, KTRs, HCPs, researchers, and review of published literature (41). Feasibility outcomes and progression criteria are found in Table 1 below. In addition, the fidelity of the ExeRTiOn DHI was assessed.

**Table 1:** Feasibility outcomes and a priori progression criteria.

**Criteria**	**Pre-set cut offs**
Screening of potential participants	≥50% deemed eligible approached to do the study consider progression to a definitive trial If <50% and no significant valid reasons provided, consider not progressing to a further study
Recruitment rate	≥50% consider progression to a definitive trial 40–49% TMG to discuss trial, and if valid modifiable reasons identified, the study may progress ≤ 30% and there are no significant valid reasons provided, the study will not progress to a definitive trial
Retention rate at 12-months	≥60% progress research 50–59% discuss with TMG. If valid reasons identified, the study may progress ≤ 40% do not consider further research
Intervention adherence	≥60% of the intervention completed (≥7 out of the 12 sessions) If <60% adherence, with no valid reasons from discussions with the TMG, the study may not progress
Safety and hospitalizations	Capture and report any harms e.g., Slips/trips Capture and report unplanned hospitalizations Capture and report any associated adverse events Non-related serious adverse events were defined as unplanned and unrelated hospitalizations (≥24 h).

*TMG, trial management group*.

### Secondary Outcomes

Anthropometric measures included body weight (kilograms) waist circumference (centimeters), hip circumference (centimeters) and BMI (kg/m^2^). Blood pressure and heart rate were recorded three times on each occasion and averaged. Bioimpedance analysis was assessed using the Fresenius body-composition Monitor (Fresenius BCM) (42, 43), a CE marked device (44). Fat mass and lean tissue mass were recorded.

Functional exercise capacity was assessed by the 6-minute walk test, using a standardized protocol (45). Pre and post resting heart rate, and total 6-minute walk distance (meters) was recorded.

Arterial stiffness was measured by pulse wave velocity and augmentation index, using the Vicorder system (Skidmore Industries, UK). Standardized procedures (46) and calculations of arterial path length (47) were used. Pulse wave velocity and augmentation index were measured three times, and then averaged for a final score.

A number of questionnaires were completed at each study visit. The General Practice Physical Activity Questionnaire which has been validated in people living with kidney disease (48) classified PA into four categories: inactive, moderately inactive, moderately active and active (49). The Nutrition Self-Efficacy Scale and the Physical Exercise Self-Efficacy Scales (50) assessed self-efficacy. Higher scores indicated greater likelihood to change the targeted behavior (e.g., PA) (50). The Euro-QOL 5-Dimension-5-level questionnaire (EQ-5D-5L) (51), which has been validated in KTRs as a measure of health status (52), assessed health-related quality of life. The EQ-5D-5L visual analog scale, and the EQ-5D-5L index value were collected (53). The EQ-5D-5L index value was calculated using the van Hout et al. (54) method, using a downloadable calculator (55). Fatigue symptom severity was assessed by the Chalder Fatigue Scale (56), which included sub-scales for physical and mental fatigue, and a total fatigue score (from 0 to 33) (56). Permission was obtained to use the Chalder Fatigue Scale and EQ-5D-5L. Participant and transplant characteristics were collected from medical records. Estimated glomerular filtration rate (eGFR) was calculated using the CKD-EPI creatinine equation (measured in ml/ min/1.73m^2^) (57), and the CKD-EPI calculator (58). Serum creatinine blood results (μmol/L) from routine transplant clinic blood tests that were conducted on the same day as the study visits were used.

### Sample Size

As recommended by the Consolidation of Reporting Trials (CONSORT) guidelines for feasibility trials (59), formal power calculations were not completed. The initial target sample was 50 participants. A sample size between 24 and 50 has been recommended to estimate standard deviations for use in a sample size calculation for a follow-up trial (60–62).

### Nested Qualitative Sampling

A purposive sample (63) of trial participants were invited for individual semi-structured interviews to explore the experiences of participating in the trial, and the experiences using the ExeRTiOn DHI. To capture both groups experiences regarding the feasibility of taking part in this trial, participants from both groups were sampled for the qualitative interviews. A range of age, gender, and adherence with the DHI were included in the qualitative sampling framework. The final qualitative sample size was informed by the inductive reflexive analysis (64), information power (65), and the meaning and themes derived from the analysis (66). *A priori* analysis estimated sample size of 5 to 10 rich interviews would be sufficient to uncover common patterns and themes from across the dataset.

### Statistical Analysis Plan

Since this was a feasibility study (59), no significance testing was performed. Descriptive statistics are presented with corresponding two-sided 95% confidence intervals using SPPS© for Mac (Version 27). Summary statistics were presented using Medians and interquartile ranges (IQRs). Analysis followed the intention-to-treat principle i.e., all participants with a recorded outcome were included in the analysis according to the treatment group to which they were randomized regardless of treatment actually received.

All qualitative interviews were recorded, transcribed, and imported into NVIVO for MAC© Version 12 for analysis. Data quality and richness was assessed using information power (65). Reflexive thematic analysis (64, 67), from a pragmatic philosophical standpoint (68) was performed.

A convergent mixed-methods analysis was used (69). Joint display tabulation sought examples of convergence, complementary issues or discrepancies between the qualitative and quantitative datasets (70).

## Results

### Feasibility Outcomes

#### Eligibility, Screening, and Recruitment

**Figure 1:**
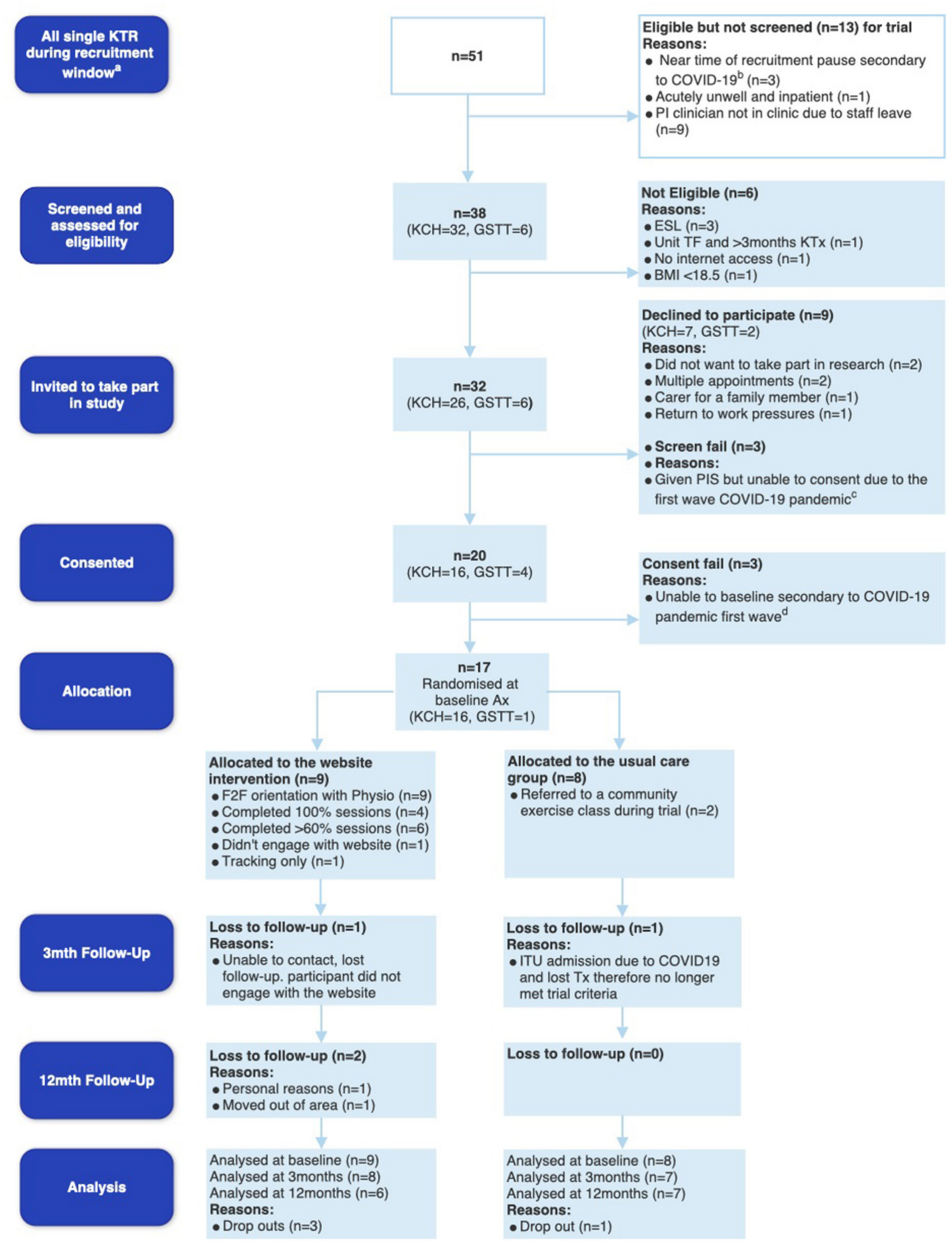
Feasibility CONSORT diagram. ^a^Indicates the recruitment window (3rd September 2019–15th March 2020 for KCH and 19th February−15th March 2020 for GSTT), ^b^indicates potential participants at KCH who were eligible days before recruitment was put on hold due to Coronavirus disease 2019 (COVID-19) on the 15th March 2020, ^c^demonstrates the 3 potential participants at KCH who were given patient information sheets but unable to consent due to the first wave of COVID-19, and ^d^indicates 3 participants who consented at GSTT but unfortunately due to pausing of recruitment, became ineligible and were therefore not baselined or randomized. KTR, kidney transplant recipients; PI, Principal Investigator; KCH, King's College Hospital; GSTT, Guy's and St Thomas' Hospital; ESL, English as a second language; TF, transfer; BMI, body mass index; ITU, Intensive Care Unit.

Recruitment of participants took place from the 3rd of September 2019, was paused on the 15th of March 2020, when 20 participants had been recruited, and then ceased on the 2nd of June 2020 due to the COVID-19 pandemic, the shielding of KTRs and the cessation of kidney transplant surgeries in the UK. An amendment to ethics was submitted and approved on the 6th of August 2020. Figure 1 below depicts the feasibility CONSORT diagram (59).

Whilst there were 51 new KTRs within the trial recruitment period, *n* = 13 were not screened due to acute illness of potential participants, staff leave of the principal investigator completing recruitment, and some participants being identified right before the recruitment was halted due to the outbreak of COVID-19 (see Figure 1). Of the 38 new KTRs screened, 32 were eligible for the study with a screening rate of 84.2% (95% CI 68.6 to 94.0%). Twenty consented to the trial, with a consent rate of 62.5% (95% CI 43.7 to 79.0). Reasons for declining participation included multiple hospital appointments (*n* = 2), declining research participation (*n* = 2), caring for family members (*n* = 1), and return to work pressures (*n* = 1). Unfortunately, 3 participants who consented, were unable to complete baseline assessment and randomization (consent fails). Seventeen participants completed baseline assessments and were randomized to UC (*n* = 8) or the DHI IG (*n* = 9) (Figure 1).

#### Participant Characteristics

Of the 17 participants, 10 were male (58.8%), with a median age of 49 (IQR 39.6) years. The median transplant vintage was 62 days (IQR 53.0, 68.0). Table 2 demonstrates the baseline participant characteristics.

**Table 2:** Participant characteristics at baseline.

**Variable**		**Total (*n =* 17)**	**Intervention group (*n =* 9)**	**Usual care (*n =* 8)**
Age	Years, median (IQR)	49.0 (39.0 to 59.0)	39.0 (33.0 to 44.0)	59.5 (53.5 to 65.0)
Sex	Males, *N* (%)	10 (58.8%)	5 (55.6%)	5 (62.5%)
Ethnicity	White Caucasian, *N* (%)	6 (35.3%)	3 (33.3%)	3 (37.5%)
	Black African and Caribbean, *N* (%)	9 (52.9%)	5 (55.6%)	4 (50%)
	Asian, *N* (%)	2 (11.8%)	1 (11.1%)	1 (12.5%)
Post-transplant time	days	62.0 (53.0 to 68.0)	62.0 (58.0 to 79.0)	59.0 (49.5 to 66.50)
Donor type	Live related, *N* (%)	2 (11.8%)	1 (11.1%)	1 (12.5%)
	Live unrelated, *N* (%)	2 (11.8%)	1 (11.1%)	1 (12.5%)
	Deceased, *N* (%)	13 (76.5%)	7 (77.8%)	6 (75.0%)
Two or more previous KTx	*N* (%)	4 (23.5%)	3 (33.3%)	1 (12.5%)
Episodes of acute rejection	*N* (%)	4 (23.5%)	2 (22.2%)	2 (25.0%)
CKD diagnosis	GN, *N* (%)	7 (41.2%)	5 (55.6%)	2 (25.0%)
	DN, *N* (%)	2 (11.8%)	1 (11.1%)	1 (12.5%)
	HT, *N* (%)	2 (11.8%)		2 (25.0%)
	Other and unknown, *N* (%)	6 (35.3%)	3 (33.3%)	3 (37.5%)
RRT before KTx	Pre-emptive transplant, *N* (%)	1 (5.9%)		1 (12.5%)
	HD, *N* (%)	10 (58.8%)	6 (66.7%)	4 (50%)
	PD, *N* (%)	3 (17.6%)	1 (11.1%)	2 (25%)
	HD and PD, *N* (%)	3 (17.6%)	2 (22.2%)	1 (12.5%)
RRT duration pre KTx	Months, median (IQR)	34.0 (24.0 to 58.0)	37.0 (34.0 to 58.0)	30.0 (22.5 to 52.0)
Baseline body weight	Kilograms, median (IQR)	92.6 (72.0 to 96.1)	94.5 (63.0 to 102.0)	81.3 (73.6 to 94.6)
Baseline BMI	kg/m^2^, median (IQR)	27.9 (23.9 to 32.9)	30.0 (23.9 to 33.6)	26.8 (24.6 to 29.8)
Immunosuppression regime (total daily dose)	Tacrolimus, median (IQR)	16.0 (8.0 to 20.0)	16.0 (10.0 to 20.0)	13.0 (6.0 to 24.0)
	Prednisolone, median (IQR)	5.0 (5.0 to 7.5)	5.0 (5.0 to 5.0)	8.8 (5.0 to 10.0)
	Mycophenolate Mofetil, median (IQR)	1,000 (1,000 to 1,000)	1,000 (500 to 1,000)	1,000 (1,000 to 1,000)
Baseline renal function (mL/min/1.73 m^2^)	CKD-EPI creatinine eGFR, median (IQR)	40 (32 to 60)	42.0 (29.0 to 64.0)	40.0 (33.0 to 44.0)
Smoking history	Current smoker, *N* (%)	2 (11.8%)	1 (11.1%)	1 (12.5%)
	Ex-smoker, *N* (%)	6 (35.3%)	3 (33.3%)	3 (37.5%)
Anti-hypertensive medications	Taking antihypertensives, *N* (%)	11 (64.7%)	7 (77.8%)	4 (50.0%)
	Number of antihypertensive medications, median (IQR)	1.0 (0.0 to 1.0)	1.0 (0.1 to 1.0)	0.5 (0.0 to 1.0)
Baseline blood pressure (mmHg)	SBP, median (IQR)	138.0 (121.0 to 149.0)	137.0 (121.0 to 148.0)	143.0 (117.5 to 150.0)
	DBP, median (IQR)	83 (73.0 to 88.0)	83.0 (73.0 to 86.0)	85.5 (75.0 to 90.5)
Diabetes diagnosis	Type 1 diabetes, *N* (%)	1 (5.9%)	1 (11.1%)	
	Type 2 diabetes, *N* (%)	2 (11.8%)		2 (25%)
	PTDM, *N* (%)	1 (5.9%)	1 (11.1%)	
Diabetic medication	Insulin only, *N* (%)	3 (17.6%)	2 (22.2%)	1 (12.5%)
Number of comorbidities^*^	One, *N* (%)	9 (52.9%)	6 (66.7%)	3 (37.5%)
	Two or more, *N* (%)	8 (47.1%)	3 (33.3%)	5 (62.5%)

*Median and IQR ranges (IQR) are presented for continuous data. Proportion percentages and frequency numbers are shown for categorical data*.

* *Indicates comorbidities included a medical history of diabetes, hypertension, cerebrovascular event, osteoarthritis, brain hemorrhage, cardiovascular disease, cancer or respiratory disease. Episodes of acute rejection were classified as yes or no within the first 3 months from medical notes and biopsy reports. CKD, chronic kidney disease; KTx, Kidney Transplant; GN, glomerular nephritis; DN, Diabetic Nephropathy; HT, Hypertension cause; RRT, Renal replacement therapy; HD, hemodialysis; PD, peritoneal dialysis; PTDM, post-transplant diabetes mellitus; BMI, body mass index; eGFR, estimated glomerular filtration rate; BP, blood pressure; SBP, systolic blood pressure; DBP, diastolic blood pressure*.

The median eGFR (IQR) was 40 (32 to 60), 43 (40 to 58.5) and 52 (33 to 66) (mL/min/1.73 m^2^). Most participants were prescribed triple immunosuppressant regime at baseline (Tacrolimus, Prednisolone and Mycophenolate Mofetil) (Table 2). The median total daily dose of mg of Prednisolone was maintained throughout the trial. At baseline, only one IG participant had a diagnosis of post-transplant diabetes mellitus. Supplementary Material 1 depicts detailed sample characteristics.

#### Retention

Four out of the 17 participants that were randomized did not complete the trial (IG *n* = 3, UC *n* = 1). The total sample 12-month retention rate was 76.4% (95% CI 50.0 to 93.2). The IG 12-month retention rate was 66.7% (95% CI 29.2 to 92.5). The UC 12-month retention rate was 87.5% (95% CI 47.4 to 99.7%). Withdrawal reasons are depicted in Figure 1.

#### Adherence to the DHI

Adherence with the 12-weekly sessions varied. The median number of total sessions completed by IG participants was 10 (IQR 5 to 12) out of the 12-weekly sessions (Table 3). Six out of the nine IG participants (66%, 95% CI 29.9 to 92.5%) met the progression criteria of adhering to 60% or more of the sessions. Four participants completed all 12 sessions. Three participants were partial completers and had individual adherence rates of 75, 42, and 83%, respectively. One IG participant chose to only use the body weight and PA tracking functions of the website, and but did not complete the 12-weekly sessions. Another IG participant chose not to engage with the website and was lost to follow-up (Figure 1). “Trigger messages” were activated in 7 participants, with 2 participants re-engaging with and completing the 12-week ExeRTiOn DHI. Three IG participants chose to re-visit the ExeRTiOn DHI after completion of the sessions to review content *(n* = 2) or continue with the physical activity and body weight tracking function (*n* = 1). Six of the nine IG participants (66.7%) chose to view the ExeRTiOn DHI with their smart phones (see Table 3). Table 3 below summarizes IG participants engagement with the ExeRTiOn DHI.

**Table 3:** Intervention group participants engagement with the ExeRTiOn DHI.

**Variable**		**IG participants (*n =* 9)**
Devices used to view the ExeRTiOn	Smartphones	6 (66.7)
DHI, *n* (%)	Tablet	1 (11.1)
	Laptop	1 (11.1)
	PC	1 (11.1%)
Number of logins to the ExeRTiOn DHI,		13 (7 to 22)
median (IQR)		
Number of sessions completed, median (IQR)		10 (5 to 12)
Minutes to complete session 1, median (IQR)		5 (1 to 10)
Minutes to complete session 2, median (IQR)		9 (6 to 16)
Minutes to complete session 3, median (IQR)		6 (5 to 12)
Minutes to complete session 4, median (IQR)		7 (4 to 8)
Minutes to complete session 5, median (IQR)		9 (8 to 10)
Minutes to complete session 6, median (IQR)		4 (2 to 19)
Minutes to complete session 7, median (IQR)		7 (6 to 7)
Minutes to complete session 8, median (IQR)		4 (1 to 6)
Minutes to complete session 9, median (IQR)		3 (1 to 5)
Minutes to complete session 10, median (IQR)		9.5 (5 to 17)
Minutes to complete session 11, median (IQR)		18 (7 to 20)
Minutes to complete session 12, median (IQR)		12 (8 to 19)
Total per-participant physical activity minutes entered		650 (250 to 1736.0)
into DHI, median (IQR)		
Number of goals set per participant, median (IQR)		3 (1 to 5)
Type of goals set on the ExeRTiOn	PA only	2 (22.2%)
DHI, *n* (%)	diet only	1 (11.1%)
	both PA and diet	4 (44.4%)
	no goals set	2 (22.2%)

*Continuous data summarized using Median and IQR. Categorical data is shown using proportions (n, %). IG, intervention group; DHI, digital health intervention; IQR, interquartile range; PA, physical activity*.

#### Fidelity of the ExeRTiOn DHI

The ExeRTiOn DHI was retrospectively mapped to the behavior change wheel (BCW) (26, 27) and coded to the behavior change technique taxonomy version 1 (BCTTv1) (25). All physiotherapist encounters were anonymized and coded in NVIVO, refer to Supplementary Material 2. ExeRTiOn content was read and re-read and coded using a BCTTv1 coding framework (27). Whilst BCT's known to inform PA and healthy eating behaviors (24) were central to the design and development of the ExeRTiOn DHI (16), *post-hoc* coding revealed 11 additional BCT's.

The most frequently represented BCT in the ExeRTiOn DHI was BCT “prompt and cues” (25) that was used 25 times. These in-built prompts occurred throughout each of the 12-weekly sessions and facilitated participant engagement with the ExeRTiOn DHI. The most frequent BCT in the physiotherapist interactions was BCT “social support (unspecified)” (27) which was used 83 times. This included advice, praise, and encouragement throughout the personalized messages. “Social support (unspecified)” was thought to influence each of the three target behaviors of the ExeRTiOn DHI (Increase PA, engagement with the ExeRTiOn DHI, and the use of a balanced diet (including healthy eating and portion control).

#### Outcome Acceptability

Assessment visits with recruited participants took place from the 27th of September 2019 to the 22nd of March 2021. The median time to complete assessments at baseline, 3- and 12-months was 70 min (IQR 60 to 88) (*n* = 17), 48 min (IQR 30 to 60) (*n* = 15), and 50 min (IQR 48 to 53) (*n* = 13). There were no missing data at baseline. Missing data at 3- and 12-months was due to study dropouts (*n* = 4), and the challenges associated with conducting research in an extremely clinically vulnerable population during the COVID-19 pandemic. At 3-months 8 participants were unable to complete full outcomes due to shielding during the fast wave of the COVID-19 pandemic, with 6 assessments being conducted over the telephone. Therefore, face-to-face outcomes (bioimpedance analysis, pulse wave velocity, augmentation index, waist- and hip-circumference, and 6-minute walk test) were not collected. Clinical data (bloods, body weight, blood pressure and heart rate) were collected from medical records. Questionnaires and qualitative interviews were conducted over the telephone. At 12-months, 12 out of the 13 participants completed face-to-face assessment with COVID-19 safety procedures *in situ*. One participant (UC) requested a virtual follow-up.

#### Safety and Hospitalizations

There were no associated serious adverse events. Six non-related serious adverse events occurred evenly across the study sample (*n* = 3 IG, *n* = 3 UC group). Reasons included hospitalization for COVID-19 (*n* = 1 UC, *n* = 1 IG), urgent transplant renal artery angioplasty (*n* = 1), elevated blood glucose levels due to post-transplant diabetes mellitus (*n* = 1), an episode of Cytomegalovirus viraemia (*n* = 1) and acute transplant rejection (*n* = 1). Unfortunately, a UC participant lost their transplant during intensive care admission for COVID-19 and were withdrawn from the trial (Figure 1). Seven participants had an episode of transplant rejection confirmed via biopsy. Ten (5 from each group) experienced Cytomegalovirus viraemia requiring treatment with valganciclovir.

### Secondary Outcomes

**Figure 2:**
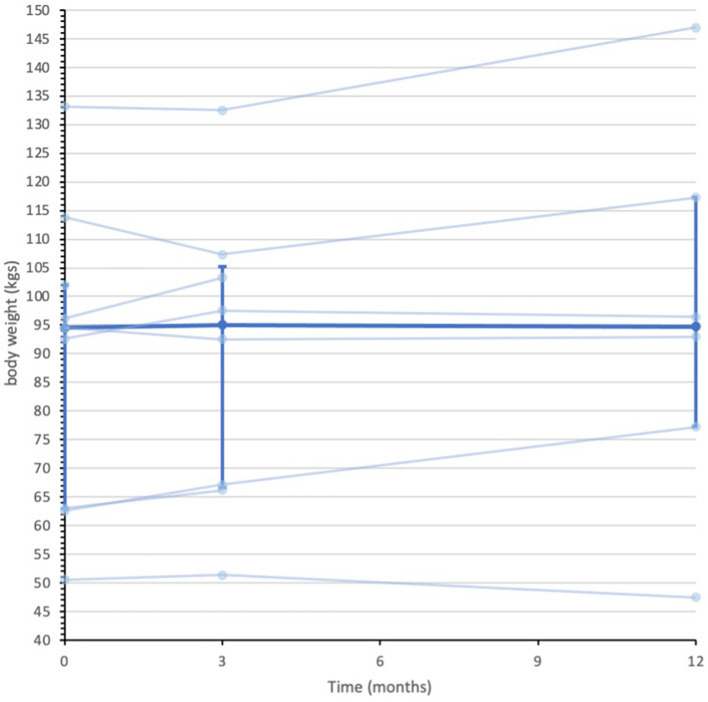
Data series of individual and median body weight values for IG participants (*n* = 9). Individual data series for participants in the intervention group depicted by the pale blue lines. Median depicted by darker blue line, with IQR error bars. Median was calculated from all recorded data at each assessment point. *n* = 9 at baseline, *n* = 8 at 3-months, and *n* = 6 at 12-months.

**Figure 3:**
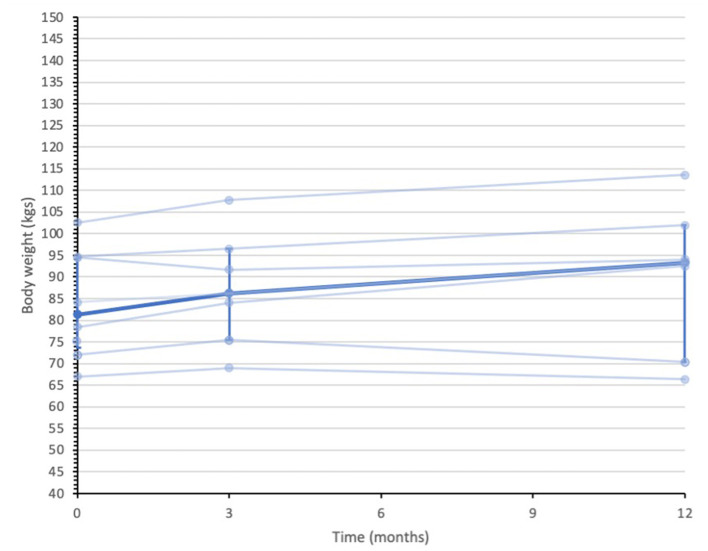
Data series of individual and median body weight values for UC participants (*n* = 8). Individual data series for participants in the usual care group are depicted by the pale blue lines. Median depicted by darker blue line, with IQR error bars. Median was calculated from all recorded data at each assessment point. *n* = 8 at baseline, *n* = 7 at 3-months, and *n* = 7 at 12-months.

Secondary quantitative outcome data are summarized in Supplementary Material 3. Median (IQR) IG bodyweight were 94.5 (63.0 to 102.0) kilograms (kgs) at baseline, 95.0 (66.7 to 105.3) kgs at 3-months and 94.7 (77.2 to 117.3) kgs 12-months. In contrast, the UC group median (IQR) body weight measures were 81.3 (73.6 to 94.6) kgs at baseline, 86.2 (75.4 to 96.5) kgs at 3-months and 93.3 (70.3 to 101.9) kgs at 12-months. Figures 2, 3 display individual and median body weight values for both groups.

Median 6-minute walk distance (IQR) measurements were 450 (450 to 540) meters (m) at baseline, 525 m (472.5 to 615 m) at 3-months, and 495 m (465 to 615 m) at 12-months in the IG. In the UC group, the median 6-minute walk distance (IQR) were 517.5 m (436 to 570 m) at baseline, 507.5 m (442.5 to 605 m) at 3-months, and 435 m (435 to 555 m) at 12-months. Median BMI, waist- and hip-circumference, pulse wave velocity, augmentation index, and questionnaires appeared comparable across the sample (see Supplementary Materials 3, 4).

### Qualitative Results

Thirteen participants were invited to and completed individual semi-structured interviews between the 31st of January 2020 and the 20th of August 2020 (Supplementary Material 5). One interview was conducted face-to-face prior to COVID-19. The remaining 12 interviews were conducted over the telephone. Topic guides (see Supplementary Material 6) were amended to include questions regarding the impact of COVID-19.

**Figure 4:**
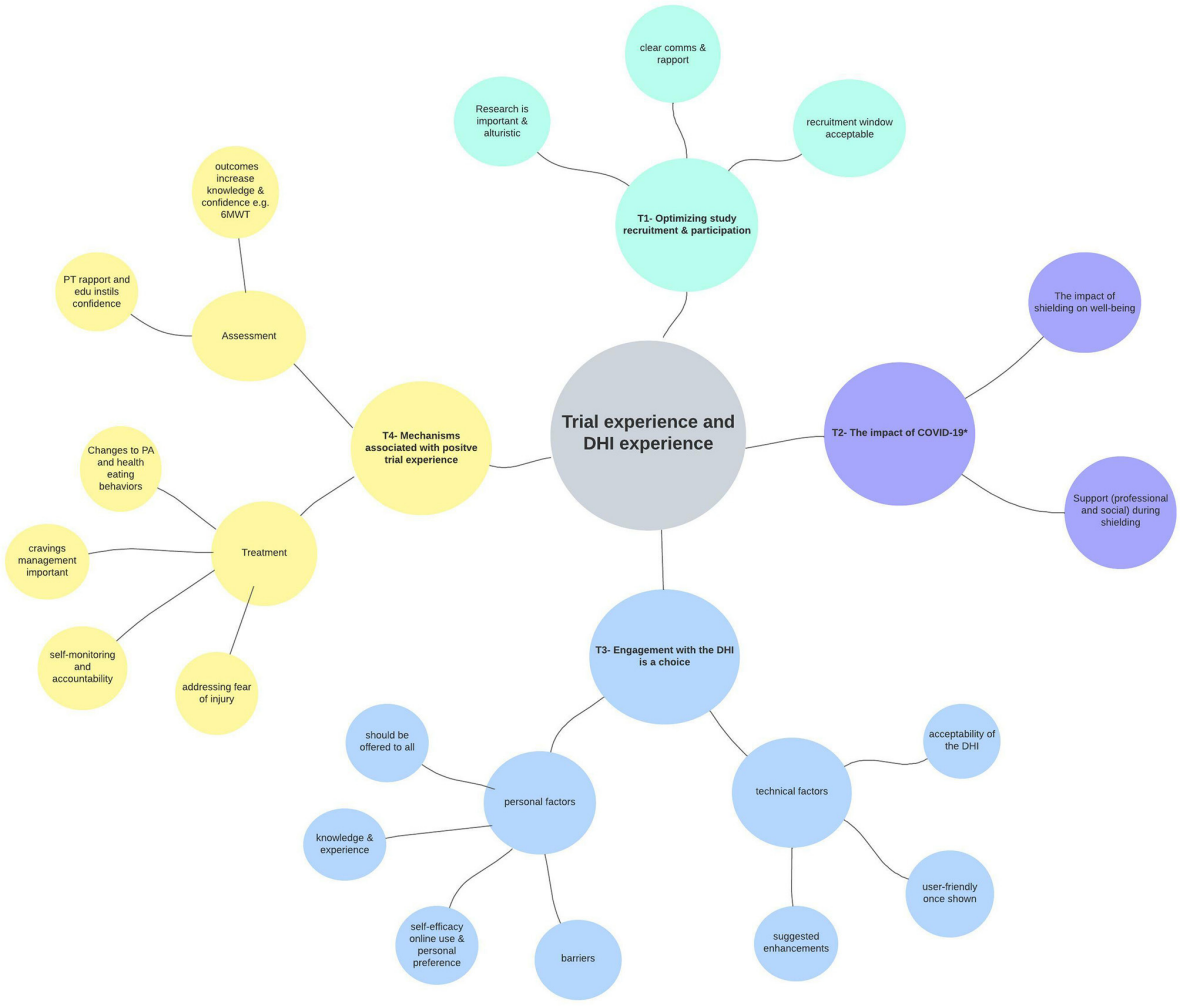
Thematic map from reflexive thematic analysis (*n* = 13). Key themes are represented in different colors. The research question is shown at the center of the diagram, with outward branching themes (T1 to T4), and subthemes from the qualitative analysis. The * in Theme 2 above depicts the first wave of COVID-19 and the shielding enforced to Kidney Transplant Recipients (23rd March 2020 to the 1st of August 2020). Ax, assessment; comms, communication; DHI, digital health intervention; edu, education; PA, physical activity; PT, physiotherapist; tech, technical; 6MWT, six-minute walk test.

Reflexive thematic analysis (44) revealed four main themes relating to the experience of using the ExeRTiOn DHI, and the experience during trial participation. Figure 4 below summarizes the final thematic map. Illustrative quotes for each theme and subtheme are depicted in Table 4.

**Table 4:** Themes relating to the acceptability, feasibility, and experience of both trial participation and the ExeRTiOn DHI with illustrative quotes.

**Subtheme/divergent quotes if evident from dataset**	**Illustrative quotes**
**Theme 1—Optimizing participant recruitment**	
Research is important and altruistic	*I am happy to do research, and you know, if it helps the next person down the line. because somebody in front has helped me. (P01, female, UC group)* *When I had one taken out, kidney taken out, I've given it straight to, I've donated it to the cancer research… because if I can help in anyway, by helping someone else, you know- all be it. (P02, male, UC group)*
Clear communication and rapport is essential	*Yeah, it was good there was no pressure, I felt like I could ask questions. Uhm, you know the paperwork I filled out was pretty self-explanatory, uhm it was very detailed you know it was very good. (P03, male, UC group)* *I think initially having that talk with physio did help me um because all you hear is hearsay quite a lot, especially when you're in the kidney clinic and talking to other participants, you're not sure who, who is being honest and who's not [laughter] but it just creates more paranoia and curiosity. (G03, IG)*
Recruitment window acceptable	*It's not an unreasonable time. And I think especially where your target people…their likely to have the time. Um. at that you know- it's not as if they're you know. they're not, especially in the first 3-months, they're not leaping around, um. Worrying about you know a busy schedule. (P10, female, IG)* *When I was recruited, I just wanted to kind of get going and basically see what the website was all about (P07, female, IG)*
Limited contrasting quotes	*I thought it was too soon. Because after the operation, I didn't even feel myself for the past- the last three- six months. (P09, female, UC group)*
**Theme 2—The impact of COVID-19**	
The impact of shielding on well-being	*It has made exercise a bit more difficult because I look after my son full-time now at home and it's hard to carve out time to exercise and I can't run in the park, I can't go to classes. So, we were doing Joe Wicks every day, but that's not the same as being outside in the fresh air exercising. (P03, male, UC group)* *I just feel like I don't want to do nothing, I can't be bothered, I just want to be left alone (P06, female, IG)*
Support (professional and social) during shielding	*I think, um it will help because I spoke to my physio quite a bit, she used to call me and um, she, she would, I'd tell her sometimes and shed be like you know what you know keep busy, do this and do that and stuff like that she would give me advice. (G03, female, IG)* ***Interviewer:** Has there been anything else COVID-19 has made it harder for you to do, in regard to the trial?* ***P07:** uhm no, because like I said I can access it on my phone that I have with me, so no. (female, IG)*
**Theme 3—Engagement with the ExeRTiOn DHI is a choice**	
Personal factors	*We are both [partner] definitely working slightly longer for working from home. And therefore, you feel tired from a different way. (P15, male, IG) She [research fellow] helped me go to the website, and in the beginning, I actually forgot about going to the website because I uh wasn't used to, so she actually reminded me sometimes to go and do my exercise. (P05, male, IG)* *I was just [pause] following the programme through.. Um but that was just my personal thing. Just because I have- you know I have the knowledge and the confidence to do my own thing. (P10, female IG)* *It would be cool to know that is the kind of thing that is presented to people once they have had a transplant. Because there are going to be people who are in worse positions then me… I think it would be good to give them the option. Because it's always nice to have the option to do this (P15, male, IG)*
Technical factors	*The problem-solving thing, um there was steps where it said identify the problems…it was a bit too much, there was a lot of things that you had to write down (GO3, female, IG)* *Maybe under different tabs for example- different link or tab. This is for older people with less strength. And then for I don't know, younger participants? Because I have seen some there was some transplant participants (hospital name), they are younger. They can lift more whilst they recover (P12, male, IG)* *I would say instead of pictures, maybe get videos, uhm but I think there is a video where there is instructing, what sort of exercise would look like (GO3, female, IG)* *Like the website was straight forward and the videos explained anything that if I'd had queries to, the videos would answer it. (P07, female, IG)*
Limited contrasting quotes	*I think if it was something more like [pause], let me see, in a group or more personal thing, think like not on the phone you get into a group to do the exercises it would be more motivating to do it (P06, female, IG)* *If I'm honest I don't think there is much of a change in my opinion. I found there was far much more on that site erm that I even needed.. you know rather than going on the internet, rather than going on you know other websites and stuff I found that this particular website that there was a lot on there to help. (P04, male, IG)* *To navigate around it, I found, I found it a bit difficult at first, I didn't really get it. (P06, female, IG)* *The more you use it, the more you get used to it, so then it is not so bad... I realized that if I just give it go, then I would be able to do it. (P06, female, IG)*
**Theme 4—Mechanisms of action associated with a positive study experience**	
Assessment factors	*Yeah. I think that one was good. Because [pause] we need these things to check if everything is working well in our life. So yeah. I think it helped. It put my mind- it give me piece of mind. (P09, female, UC group)* ***Interviewer**: what your overall experience of this research trial has been like for you?* ***P01** excellent. excellent. It has shown me that I can walk. If I put my mind to it [laughs]... really walk. (P01, female, UC group)* *It was just the conversation you're having whilst you're doing the trial uhm, I think makes you a lot more at ease anyway. Like [physio name]*
	*[pause] you know talking to her like she was my sister sort of thing not as like a doctor. You know yeah it made you feel very comfortable. (P02, male, UC group)* *I was more muscles than my fat because I was very worried about the fat. but when she-she measured the muscles within me and the fat she told me that I was more muscles than the fat I was thinking of. She-she even went ahead to tell me about the percentage of muscles that I had so I was very very uh-u-h I actually felt very good. (P05, male, IG)*.
Treatment factors	*If I do exercise, what if I damage my new kidney, that's the only thing that comes to your mind...: but when I saw the exercises on there, it was very much um, you know puts you at ease and you know, you knowing that it's not anything that is going to hurt you physically. (G03, female, IG) When I started, I had pains in my abdomen, but gradually it went away, as I began to exercise. (P05, male, IG)* *What the exercise on the website does is, is quite um almost like a baby step kind of thing, like it is all up to your pace, it's all up to um what pace you can do, and I think the more active you have become, the more you can go faster, the more you can do extra steps or anything like that, so without it I don't think I would have like you know recovered as fast as I did. (G03, female, IG)* *To learn about the-the-the exercise, yes about the exercise, so I go there to remind myself about the exercise and-and-and the cravings. And-and sometimes I-I show the uh food the proportion to my wife and telling her that and I need to eat more vegetables and fruits than the carbohydrate. (P05, male, IG)* *It [DHI] made me do exercise, for someone who doesn't like exercise at all, uhm [laughter] it made me at least do 10 minutes a day, because obviously I have the kids and now that they are not in school so at least taking 10 minutes out of my day, to do that. I've actually started to do that, and it's been a thing I have been doing since so that's helped (P07, female, IG)* *With the tracking your weight and you're exercising, or you know your activities through your day or your week. I found by keeping a track of it kind of motivates you to want to add more to the activity part, and then to the part where you've got the weight, your-I mean for myself as well I look at it and I'm like you know I want to try and bring that weight down down down. (P04, male, IG)* *So, my point there is in terms of being accountable to something. Even though it's not a- a human being, you are being accountable to a system, and you know-you know for these 12 weeks, you need to you know, every week you need to be putting the inputs in [weight and activity tracker]. (P10, female, IG)* *It's helped me to make better choices when I eat, or I was having problems with craving at first. But when I watched that video on how to manage cravings that was helpful. So I'd say that one, that one stood out, I forgot about that one, that one stood out, that video (P06, female, IG)*
Limited contrasting quotes	*I don't want to sort of overwork it and end back up at stage 1 again. (P02, male, UC group)* *It was a big wound. It was really, paining. and it maybe could affect your kidney. Because I don't know how the kidney. I don't to shake the kidney, I don't want anything to go wrong, so I take it easy. So that that was why you know taking it easy. Not to do stress there. Serious exercise, or shaking myself, or doing something worse, just taking it easy. (P09, female, UC group)*

*UC refers to usual care group participants, IG, intervention group participants, P and G, to participant numbers*.

#### Theme 1- Optimizing Participation and Recruitment

Research participation was seen as an important opportunity to “give back” to the community after receiving the “gift” of a kidney transplant. This altruistic view was consistently associated with reports of fostering research participation. Clear written and verbal communication, and rapport with the research staff aligned with a positive recruitment experience. The ability to ask questions and seek answers from a specialist physiotherapist was perceived as an important source of information. Largely, the recruitment within 3-months of transplantation was acceptable. However, one participant felt that recruitment window was too short (Table 4).

#### Theme 2- the Impact of COVID-19

The breath and severity of COVID-19 was consistently reported across the dataset. Shielding measures were viewed to have had a direct impact on physical and mental well-being. Unique barriers were presented by participants who were shielding at home and influenced PA behavior and motivation. “Trigger messages,” sent by the trial physiotherapist were identified as a tool to navigate personal barriers such as time, work, challenges arising from COVID-19, and to support participants to re-engage with the DHI. Support (from families and professionals), mental resilience, and a positive mindset were frequently reported as facilitators to navigate the unique challenges experienced by the KTRs that arose from the shielding measures in place during the outbreak of COVID-19 (Table 4).

#### Theme 3- Engagement With the DHI Is a Choice

Engagement with the ExeRTiOn DHI was described as an individual choice, influenced by both personal and technical factors (Figure 4). Previous knowledge and experience of PA and healthy eating behaviors, preference for mode of delivery of the weight gain prevention and self-efficacy appeared to be linked with self-efficacy in this dataset. The ExeRTiOn DHI was suggested as a flexible mode to deliver interventions in the acute recovery phase of kidney transplantation (Table 4).

The brief one-on-one orientation session at the start of the intervention with the trial physiotherapist was widely reported as essential. Some participants felt the DHI was easy to use whilst others felt some enhancements could be considered. For example, it was suggested to reduce the length of activities within session 10 (overcoming barriers) and session 11 (problem solving). Participants also suggested the “home exercise diary tab” could be categorized into different functional abilities, and the addition of a virtual group exercise class could facilitate motivation and engagement. One participant reported initial difficulty with the DHI. However, this improved with repeated use. Overall, participants felt that the ExeRTiOn DHI was acceptable, and provided a supportive space for new KTRs to address PA and healthy eating behaviors after kidney transplantation (Table 4).

#### Theme 4- Mechanisms of Action

The face-to-face study visits were viewed as a key mechanism and were consistently aligned with a positive study experience. Participants apportioned value to the opportunity to have an additional “check-up” and “benchmark” their functional abilities. The 6-minute walk test and bioimpedance analysis were the most valued outcomes. The completion of the 6-minute walk test acutely post-transplant with the trial physiotherapist was suggested to enhance confidence in walking ability, irrespective of group allocation.

Participants in the DHI group reported both changes to PA and healthy eating behaviors, with session 2 (management of cravings) being the most valued session. Self-monitoring and monitoring and feedback by the trial physiotherapist were suggested to be associated with accountability and could encourage engagement with the ExeRTiOn DHI. In contrast, participants from the UC group reported little to no difference in PA and healthy eating behaviors.

The fear of injuring the new kidney was widespread in this dataset. IG participants viewed the ExeRTiOn DHI as “baby steps” or “steppingstones” to build up PA after surgery. This gradual approach was described as a potential mechanism for the ExeRTiOn DHI to improve PA behavior and confidence. In contrast, participants in the UC group reported that they didn't want to “push-it” with PA after kidney transplantation. Data describing limited changes in PA activity largely originated in data from UC participants.

The ability to access “expert” advice and social support by the trial physiotherapist through the secure message function was seen to further enhance the positive DHI experience. A Consistent report from all interview participants, irrespective of randomization, was that the DHI should be offered to all new KTRs post-surgery (Table 4).

### Integrated Mixed Methods Analyses

The integration of qualitative and quantitative results suggests that an RCT using the ExeRTiOn DHI is feasible and acceptable for new KTRs. Further studies should ensure there is clear communication and rapport with researchers and valued patient assessment outcomes (e.g., 6-minute walk test and bioimpedance analysis are included). Craving management, self-monitoring of PA and body weight, monitoring and social support (unspecified) by the trial physiotherapist, and gradual PA were identified as factors that could have contributed to the success of the DHI.

## Discussions

The primary feasibility outcomes achieved in this study were a screening rate of 84.2% (95% CI 68.8 to 94.0), a consent rate of 62.5% (95% CI 43.7 to 79.0%), 12-month retention rate of 76.4% (95% CI 50.0 to 93.0), adherence rate to baseline assessment of 100% (95% CI 80.5 to 100.0), 3-month assessment of 88.3% (95% CI 63.6 to 98.5), 12-month assessment of 76.4% (95% CI 50.0 to 93.2%), and an adherence rate to the ExeRTiOn DHI of 66.7% (95% CI 28.9 to 92.5). There were no associated adverse events, and 29.4% of participants had a non-related adverse event.

Despite the outbreak of COVID-19 during this study, all *a priori* progression criteria were achieved. Table 5 below demonstrates the mixed-methods results against the feasibility outcomes and progression criteria. The 12-month retention rate of 76.4% from this study exceeded the progression criteria (60%) and was comparable to previous face-to-face exercise interventions in people living with chronic kidney disease (71). Adherence rates to study visits were satisfactory despite the COVID-19 pandemic occurring during data collection.

**Table 5:** Mixed-methods results against feasibility outcomes and progression criteria.

**Feasibility measure**	**Definition**	**Rates with confidence intervals**	**Progression criteria**	**Notes**
Screening rate	% Of screened participants that met the inclusion criteria during the study recruitment window	32/38 84.2% (95% CI 68.8 to 94.0)	≥50% deemed eligible approached to do the study	
Total consent rate	% Participants recruited from the total eligible potential participants in the units	20/32 62.5% (95%CI 43.7 to 79.0)	>50% of people approached consent to study who have been screened and deemed eligible to take part in the trial	Target sample of *n =* 50 not met due to changes in recruitment criteria due to COVID-19 pandemic
Trial retention at 12 months	% Participants completed trial from total sample	13/17 76.4% (95% CI 50.0 to 93.2)	Retain ≥60% of the sample at 12 months follow up	Progression criteria for retention met despite COVID-19 pandemic
Adherence to data collection at baseline Ax	% Participants who attended the baseline study visit AND completed all secondary outcomes	17/17 100% (95% CI 80.5 to 100.0)		Full outcomes include Body weight, BMI, BIA, PWV, AI, 6MWT, EQ-5D-5L, CFS, GPPAQ and self-efficacy for physical exercise and nutrition
Adherence to 3-month Ax	% Of participants who attended a 3-month assessment	15/17 88.3% (95%CI 63.6% to 98.5%)		Two participants dropped out at 3-months (one in each group)
Adherence to data collection at 3-month Ax	% Participants completing full outcome data collection at 3-months assessment from total trial sample	9/17 52.9% (95% CI 27.8 to 77.0%)		Eight participants unable to complete full assessment due to shielding during the first wave of the COVID-19 pandemic BIA, PWV, AI, waist, and hip circumference and 6MWT data were not captured
Adherence to 12-month Ax	% Of participants who attended a 12-month assessment	13/17 76.4% (95% CI 50.0 to 93.2)		Two further dropouts occurred at 12-months
Adherence to data collection at 12-month Ax	% Participants completing full outcome assessment at 12 months from total trial sample	13/17 76.4% (95% CI 50.0 to 93.2)		Participants were assessed around routine clinic visits due to COVID-19 pandemic
Adherence to the online intervention (IG only)	% Treatment group participants completing 60% (≥7/12) sessions	6/9 66.67% (95% CI 29.93 to 92.51)		6/9 participants adhered to 60% or more of the sessions Qualitative data further explored engagement
Safety and hospitalization (adverse events)	% Of participants who had a NRAE. NRAE defined as a non-elective hospital admission, of >24 h, not related to the study	5/17 29.4 (95% CI 7.8 to 51.1)	Capture and report	One participant had two NRAE's There were no related AE's
Expected and unexpected harms	Expected harms could include musculoskeletal injuries from performing exercises or slips and trips	No slips, trips or musculoskeletal injures reported	Capture and report	

*Definitions, raw numbers, proportions, and 95% confidence intervals are shown for each of the feasibility outcomes above. Willingness to be randomized is reported in the qualitative results. Ax refers to assessment, BMI, body mass index; BIA, bioimpedance analysis; PWV, pulse wave velocity; AI, augmentation index; 6MWT, six-minute walk test; CFS, Chalder fatigue scale; NRAE, non-related adverse events*.

The few existing trials utilizing exit surveys and semi-structured interviews have reported participation with other online interventions are positive and could improve accountability in KTRs (72, 73). The nested qualitative analysis in this study builds on these findings. Our interview participants postulate factors associated with a positive study experience (see Table 4 and Figure 4). The rapport with the trial physiotherapist, the education provided, and the assessment outcomes themselves such as the 6-minute walk test appeared to contribute to the acceptability of this feasibility RCT and the ExeRTiOn DHI.

The progression criteria for adherence to the ExeRTiOn DHI were satisfied, with 66% of the IG participants completing 60% or more of the 12-weekly sessions. This shows promise, given that dropout rates tend to be higher with DHI when compared with face-to-face interventions (74). Whilst other research utilizing DHI's in KTRs have reported good adherence rates (73, 75), these DHI were supported by either live video calls (73) or face-to-face sessions (75). In comparison, whilst demonstrating lower adherence rates, the ExeRTiOn DHI was completed independently, with minimal remote monitoring by the trial physiotherapist. Further studies would benefit from cost-effectiveness evaluations DHI's with minimal remote monitoring such as the ExeRTiOn DHI.

A key strength of this feasibility RCT was the involvement of KTRs throughout the design, development, and evaluation of the ExeRTiOn DHI. Prior research (10) informed iterative refinements to the ExeRTiOn product prior to this feasibility RCT. This combined intervention design approach (30), with the person-based approach (31) at the center, was thought to contribute to the acceptability of the ExeRTiOn DHI.

To our knowledge, this is the first theory-informed weight-gain prevention DHI in KTRs to be mapped to the behavior change wheel (26, 27) and coded to the behavior change technique taxonomy (version 1) (25). Online weight management interventions that include brief human interaction and personalized feedback have been shown to be clinically and statistically effective in the general population, and people living with excess weight (76–78). Qualitative data revealed the behavior change techniques social support (unspecified), goal setting behavior, self-monitoring of behavior, and outcome of behavior, were valued by our participants. Self-monitoring and goal setting are suggested behavior change techniques to promote PA and healthy eating behaviors (24).

The need for support to engage with online interventions is echoed in the few studies that explore PA and dietary combined interventions in new KTRs (15). Exit survey data from Serper et al. (72) reported participants would have valued technical support and contact with the research team. The brief personalized orientation session with the trial physiotherapist was seen as essential in this feasibility RCT, and in our previous study (10) to enhance DHI engagement. As this is a feasibility study, it was not designed to evaluate effectiveness, or the mechanisms responsible for the treatment effect. Future study design would benefit from the evaluation of what the most effective “active ingredients” and unpicking which behavior change techniques potentially mediate the treatment effect.

The management of cravings and the gradual build-up of PA to reduce fear avoidance, self-monitoring and remote monitoring by the physiotherapist were identified as valued content of the ExeRTiOn DHI. The addition of group exercise videos was suggested to improve the ExeRTiOn DHI. Similarly, Gibson et al. (73) reported KTRs participants would value the opportunity to play-back the videos to increase flexibility. Further studies would benefit from exploring delivery of educational videos to include both live and on-demand content such as kidney beam (22).

This feasibility study, by design, was not powered to detect clinically meaningful differences between groups (59). However, descriptive data on clinical outcomes such as body weight can help inform the design of future definitive studies. A reduction in 5% body weight from baseline measures is widely considered to be clinically meaningful to reduce glycaemia and cardiovascular disease risk factors (79–81). In this small sample the median body weight in the ExeRTiOn IG group from baseline to 12-months was <5% of the baseline median weight. The usual care group appeared to increase their body weight by 12 kg by the end of this 12-month feasibility study. However, adequately powered studies are required to further explore this.

The 6-minute walk test was valued by our participants to provide confidence in their functional ability in the acute post-transplant period. Booth and Adams (82) reported similar findings in a sample of advanced cancer participants completing the incremental shuttle walk test. Their participants, and family members reported increased confidence in participants functional abilities (70). The 6-minute walk test has been shown to predict mortality in other solid organ recipients (83) and be reproducible and low cost to use in children and adolescent KTRs (84).

There is no suggested minimally clinically important difference for the 6-minute walk test in KTRs. The minimally clinically important difference for the 6WMT in other populations is variably reported; 54 to 80 m in respiratory disease (85), 32 to 43, 1 m in heart failure (86, 87), and 32 m in people with multiple medical issues (88). A study in haemodialysis participants revealed that for every 100 m increase in 6-minute walk distance, there was a 5% increase in survival (89). In this current study, the IG appeared to increase their median 6-minute walk distance by 75 m from baseline to 3-months, and 45 m from baseline to 12-months. In contrast, the UC groups reduced median 6-minute walk distance by 10 m from baseline to 3-months, and by 82.5 m from baseline to 12-months. Our data suggest that the 6-minute walk test is an outcome that warrants further exploration and could provide meaningful information to KTRs and clinicians to build confidence post transplantation.

There were six non-related serious adverse events recorded in the study (3 from each group). There were no slips, trips or injuries associated with completing the ExeRTiOn DHI independently. Other studies have raised concerns for recruiting participants within the first 6 months of transplantation (73). However, this feasibility study suggests that it is possible to complete assessments and intervene safely in a sample of KTRs recruited within 3-months of transplantation.

The impact of the outbreak of COVID-19 reduced the intended sample size from 50 to 17 for this feasibility RCT, which could have influenced the validity and results. Study recruitment was prematurely halted due to the COVID-19 pandemic, which halted all non-COVID research in the UK. Due to this, and unknown timelines for when kidney transplant surgeries would resume in the UK, the Trial Management Group advised to close recruitment.

Information regarding the conduction of this trial during the COVID-19 pandemic has been transparently reported (90), and the authors accept the limitations and challenges COVID-19 had on sample size, and data collection. The reduced sample size could have explained the higher median body weight (94.5 kg vs. 81.3 kg) and age (59 years vs. 39 years) in the UC group compared to the DHI group at baseline. Moreover, it is possible that this may have influenced our findings relating to the acceptability of the DHI. Secondary outcome results warrant further exploration in a powered RCT. However, the qualitative results, and mixed-methods analysis revealed engagement with the ExeRTiOn DHI is influenced by personal factors and choice, and participants irrespective of randomization welcomed an individualized DHI to address weight gain prevention in new KTRs.

Missing outcome data was due to shielding practices resultant from COVID-19, not due to issues with the outcomes themselves. Lack of blinding could have influenced the results. Due to the nature of the study design, exercise and behavioral studies are often unable to achieve double blinding. Future follow-up studies should include blinding of the outcome assessor to improve validity. Despite these limitations, this study provides insights into future trial design. Research questions regarding the cost-effectiveness and the clinical value of the ExeRTiOn DHI across multiple sites remain unanswered. However, this was beyond the score of this feasibility RCT. Therefore, a mixed methods multi-center RCT evaluating the clinical value and cost effectiveness of the ExeRTiOn DHI is planned.

## Conclusions

This mixed-methods feasibility RCT revealed a personalized DHI for weight gain prevention after kidney transplantation was found to be feasible and acceptable to new KTRs. Despite the limitations, and the challenges faced whilst conducting research with KTRs during COVID-19, all pre-set feasibility criteria were met. Mixed-methods results provides insight into future trial design. A follow-up multi-center RCT is planned to further evaluate the clinical value and cost-effectiveness of the ExeRTiOn DHI.

## Data Availability Statement

The raw data supporting the conclusions of this article will be made available by the authors, without undue reservation.

## Ethics Statement

The studies involving human participants were reviewed and approved by the London Dulwich Research Ethics Committee (19/LO/1138). The patients/participants provided their written informed consent to participate in this study.

## Author Contributions

EC, SG, and JC conceived and designed the study. EC was involved in data acquisition. EC, JG, SG, KB, RP, and JC were involved in the statistical analyses. SG, JC, KB, and JG supervised and mentored the study. EC and SG take responsibility that this study has been reported honestly, accurately, transparently, that no important aspects of the study have been omitted, and that any discrepancies from the study as planned (and, if relevant, registered) have been explained. All authors assisted in the interpretation of data, contributed important intellectual content during manuscript drafting or revision, accepts accountability for the overall work by ensuring that questions pertaining to the accuracy or integrity of any portion of the work are appropriately investigated and resolved, and approved the final submitted manuscript.

## Funding

This work was supported by EC's Ph.D. Grant by Kidney Research UK (AHPF_001_20171122). SG was supported by the NIHR Advanced Research Fellowship (ICA-CL-2017-03-020). EC also received support from her institutions (King's College Hospital Foundation NHS Trust, and King's College London University). Fellowship grant funding included Ph.D. university fees, salary, patient travel and inconvenience fees, revisions to the ExeRTiOn DHI, hosting and tech support for the ExeRTiOn DHI from SPIKA Ltd.

## Author Disclaimer

The views expressed in this paper are not necessarily those of the NHS, the NIHR, Kidney Research UK, or the Department of Health and Social Care.

## Conflict of Interest

The authors declare that the research was conducted in the absence of any commercial or financial relationships that could be construed as a potential conflict of interest.

## Publisher's Note

All claims expressed in this article are solely those of the authors and do not necessarily represent those of their affiliated organizations, or those of the publisher, the editors and the reviewers. Any product that may be evaluated in this article, or claim that may be made by its manufacturer, is not guaranteed or endorsed by the publisher.

## References

[1] Glicklich D Mustafa MR. Obesity in kidney transplantation: impact on transplant candidates, recipients, and donors. Cardiol Rev. (2019) 27:63–72. 10.1097/CRD.0000000000000216 29870421

[2] Chan W Bosch JA Jones D McTernan PG Phillips AC Borrows R. Obesity in kidney transplantation. J Ren Nutr. (2014) 24:1–12. 10.1053/j.jrn.2013.09.002 24231063

[3] Cashion AK Hathaway DK Stanfill A Thomas F Ziebarth JD Cui Y. Pre-transplant predictors of one yr weight gain after kidney transplantation. Clin Transplant. (2014) 28:1271–8. 10.1111/ctr.12456 25159302 PMC4576829

[4] Forte CC Pedrollo EF Nicoletto BB Lopes JB Manfro RC Souza GC. Risk factors associated with weight gain after kidney transplantation: a cohort study. PLoS ONE. (2020) 15:e0243394. 10.1371/journal.pone.0243394 33370293 PMC7769456

[5] Workeneh B Moore LW Nolte Fong JV Shypailo R Gaber AO Mitch WE. Successful kidney transplantation is associated with weight gain from truncal obesity and insulin resistance. J Renal Nutr. (2019) 6:6. 10.1053/j.jrn.2019.01.009 30852120

[6] Koufaki P Greenwood SA Macdougall IC Mercer TH. Exercise therapy in individuals with chronic kidney disease: a systematic review and synthesis of the research evidence. Ann Rev Nurs Res. (2013) 31:235–75. 10.1891/0739-6686.31.235 24894142

[7] Nielens H Lejeune TM Lalaoui A Squifflet JP Pirson Y Goffin E. Increase of physical activity level after successful renal transplantation: a 5 year follow-up study. Nephrol Dial Transplant. (2001) 16:134–40. 10.1093/ndt/16.1.134 11209007

[8] Aksoy N. Weight gain after kidney transplant. Exp Clin Transplant. (2016) 14(Suppl. 3):138–40. 10.6002/ect.tondtdtd2016.P66 27805534

[9] Stanfill A Bloodworth R Cashion A. Lessons learned: experiences of gaining weight by kidney transplant recipients. Prog Transplant. (2012) 22:71–8. 10.7182/pit2012986 22489446

[10] Baker RJ Mark PB Patel RK Stevens KK Palmer N. Renal association clinical practice guideline in post-operative care in the kidney transplant recipient. BMC Nephrol. (2017) 18:174. 10.1186/s12882-017-0553-2 28571571 PMC5455080

[11] Baker RJ Mark PB Patel RK Stevens KK Palmer N. British Transplant Society Post-Operative Care in the Kidney Transplant Recipient Online. BTS (2017). Available online at: https://bts.org.uk/guidelines-standards/ (accessed December 20, 2020).

[12] KDIGO Transplant Work Group. KDIGO clinical practice guideline for the care of kidney transplant recipients. Am J Transplant. (2009) 9(Suppl. 3):S1–155. 10.1111/j.1600-6143.2009.02834.x 19845597

[13] Hricik DE. Metabolic syndrome in kidney transplantation: management of risk factors. Clin J Am Soc Nephrol. (2011) 6:1781–5. 10.2215/CJN.01200211 21734094

[14] Winters GL Kendall TJ Radio SJ Wilson JE Costanzo-Nordin MR Switzer BL. Posttransplant obesity and hyperlipidemia: major predictors of severity of coronary arteriopathy in failed human heart allografts. J Heart Transplant. (1990) 9:364–71. 2398430

[15] Castle EM McBride E Greenwood J Bramham K Chilcot J Greenwood SA. Do exercise, physical activity, dietetic, or combined interventions improve body weight in new kidney transplant recipients? A narrative systematic review and meta-analysis. Kidney Dial. (2021) 1:100–20. 10.3390/kidneydial1020014

[16] Castle EM Greenwood J Chilcot J Greenwood SA. Usability and experience testing to refine an online intervention to prevent weight gain in new kidney transplant recipients. Br J Health Psychol. (2020) 26:232–55. 10.1111/bjhp.12471 32931645

[17] Jamieson NJ Hanson CS Josephson MA Gordon EJ Craig JC Halleck F. Motivations, challenges, and attitudes to self-management in kidney transplant recipients: a systematic review of qualitative studies. Am J Kidney Dis. (2016) 67:461–78. 10.1053/j.ajkd.2015.07.030 26372087

[18] Baker L March DS Wilkinson TJ Billany RE Bishop NC Castle EM. Renal Association Clinical Practice Guideline. Exercise Lifestyle in Chronic Kidney Disease: Renal Association (2021). Available online at: https://renal.org/health-professionals/guidelines/guidelines-commentaries 10.1186/s12882-021-02618-1 PMC8862368 35193515

[19] The British Renal Society. A Multi-Professional Renal Workforce Plan for Adults and Children With Kidney Disease. (2020). Available online at: https://britishrenal.org/workforce/ (accessed June 22, 2021).

[20] Kostakis ID Kassimatis T Bianchi V Paraskeva P Flach C Callaghan C. UK renal transplant outcomes in low and high BMI recipients: the need for a national policy. J Nephrol. (2020) 33:371–81. 10.1007/s40620-019-00654-7 31583535

[21] Stauss M Floyd L Becker S Ponnusamy A Woywodt A. Opportunities in the cloud or pie in the sky? Current status and future perspectives of telemedicine in nephrology. Clin Kidney J. (2021) 14:492–506. 10.1093/ckj/sfaa103 33619442 PMC7454484

[22] Mayes J Billany RE Vadaszy N Young HML Castle EM Bishop NC. The rapid development of a novel kidney-specific digital intervention for self-management of physical activity and emotional wellbeing during the COVID-19 pandemic and beyond: kidney beam. Clin Kidney J. (2021) 15:571–3. 10.1093/ckj/sfab239 35198162 PMC8690269

[23] West R Michie S. A Guide to Development and Evaluation of Digital Behaviour Interventions in Healthcare. 1st ed. London: Silverback Publishing (2016).

[24] Michie S Ashford S Sniehotta FF Dombrowski SU Bishop A French DP. A refined taxonomy of behaviour change techniques to help people change their physical activity and healthy eating behaviours: the CALO-RE taxonomy. Psychol Health. (2011) 26:1479–98. 10.1080/08870446.2010.540664 21678185

[25] Michie S Richardson M Johnston M Abraham C Francis J Hardeman W. The behavior change technique taxonomy (v1) of 93 hierarchically clustered techniques: building an international consensus for the reporting of behavior change interventions. Ann Behav Med. (2013) 46:81–95. 10.1007/s12160-013-9486-6 23512568

[26] Michie S Van Stralen MM West R. The behaviour change wheel: a new method for characterising and designing behaviour change interventions. Implement Sci. (2011) 6:42. 10.1186/1748-5908-6-42 21513547 PMC3096582

[27] Michie S Atkins L West R. The Behaviour Change Wheel. A Guide to Designing Interventions. Great Britain: Silverback Publishing (2014).

[28] Sealed Envelope Ltd. Simple Randomisation Service. (2020). Available online at: https://www.sealedenvelope.com/simple-randomiser/v1/ (accessed August 15, 2019).

[29] Craig P Dieppe P Macintyre S Michie S Nazareth I Petticrew M. Developing and evaluating complex interventions: the new Medical Research Council guidance. BMJ. (2008) 337:a1655. 10.1136/bmj.a1655 18824488 PMC2769032

[30] O'Cathain A Croot L Sworn K Duncan E Rousseau N Turner K. Taxonomy of approaches to developing interventions to improve health: a systematic methods overview. Pilot Feas Stud. (2019) 5:41. 10.1186/s40814-019-0425-6 30923626 PMC6419435

[31] Yardley L Ainsworth B Arden-Close E Muller I. The person-based approach to enhancing the acceptability and feasibility of interventions. Pilot Feas Stud. (2015) 1:1–7. 10.1186/s40814-015-0033-z 27965815 PMC5153673

[32] Bradbury K Watts S Arden-Close E Yardley L Lewith G. Developing digital interventions: a methodological guide. Evid Based Complement Altern Med. (2014) 2014:561320. 10.1155/2014/561320 24648848 PMC3932254

[33] Bandura A. Self-efficacy: toward a unifying theory of behavioral change. Psychol Rev. (1977) 84:191–215. 10.1037/0033-295X.84.2.191 847061

[34] Miller WR Rollnick S. Motivational Interviewing. 3rd ed: Helping People Change. New York, NY: The Guildford Press (2013).

[35] MacLaughlin HL Sarafidis PA Greenwood SA Campbell KL Hall WL Macdougall IC. Compliance with a structured weight loss program is associated with reduced systolic blood pressure in obese patients with chronic kidney disease. Am J Hypertens. (2012) 25:1024–9. 10.1038/ajh.2012.80 22717545

[36] MacLaughlin HL Cook SA Kariyawasam D Roseke M van Niekerk M Macdougall IC. Nonrandomized trial of weight loss with orlistat, nutrition education, diet, and exercise in obese patients with CKD: 2-year follow-up. Am J Kidney Dis. (2010) 55:69–76. 10.1053/j.ajkd.2009.09.011 19926371

[37] Cook S MacLaughlin H Macdougall I. A structured weight management programme can achieve improved functional ability and signficant weight loss in obese patients with chronic kidney disease. Nephrol Dial Transplant. (2008) 23:263–8. 10.1093/ndt/gfm511 17977872

[38] Rollnick S Miller WR Butler C. Motivational Interviewing in Health Care: Helping Patients Change Behavior. New York, NY: Guilford Press (2008).

[39] Rollnick S Miller W. What is motivational interviewing?. Behav Cogn Psychother. (1995) 23:325–34. 10.1017/S135246580001643X 19364414

[40] Young HML Goodliffe S Madhani M Phelps K Regen E Locke A. Co-producing progression criteria for feasibility studies: a partnership between patient contributors, clinicians and researchers. Int J Environ Res Public Health. (2019) 16:3756. 10.3390/ijerph16193756 31590424 PMC6801439

[41] Harper L Morgan MD Chanouzas D Caulfield HK Coughlan L Dean C. Treatment of fatigue with physical activity and behavioural change support in vasculitis: study protocol for an open-label randomised controlled feasibility study. BMJ Open. (2018) 8:e023769. 10.1136/bmjopen-2018-023769 30377212 PMC6224747

[42] Gudivaka R Schoeller DA Kushner RF Bolt MJ. Single- and multifrequency models for bioelectrical impedance analysis of body water compartments. J Appl Physiol. (1999) 87:1087–96. 10.1152/jappl.1999.87.3.1087 10484581

[43] Macdonald JH Phanish MK Marcora SM Jibani M Bloodworth LL Holly JM. Muscle insulin-like growth factor status, body composition, and functional capacity in hemodialysis patients. J Ren Nutr. (2004) 14:248–52. 10.1016/j.jrn.2004.08.001 15483786

[44] NICE. Multiple Frequency Bioimpedance Devices to Guide Fluid Management in People With Chronic Kidney Disease Having Dialysis. NICE Guidance (2017). Available online at: https://www.nice.org.uk/guidance/dg29

[45] American Thoracic Society. ATS statement: guidelines for the six-minute walk test. Am J Respir Crit Care Med. (2002) 166:111–7. 10.1164/ajrccm.166.1.at1102 12091180

[46] Laurent S Cockcroft J Van Bortel L Boutouyrie P Giannattasio C Hayoz D. Expert consensus document on arterial stiffness: methodological issues and clinical applications. Eur Heart J. (2006) 27:2588–605. 10.1093/eurheartj/ehl254 17000623

[47] Hickson SS Butlin M Broad J Avolio AP Wilkinson IB McEniery CM. Validity and repeatability of the Vicorder apparatus: a comparison with the SphygmoCor device. Hypertens Res. (2009) 32:1079–85. 10.1038/hr.2009.154 19779487

[48] Wilkinson TJ Palmer J Gore EF Smith AC. The validity of the 'General Practice Physical Activity Questionnaire' against accelerometery in patients with chronic kidney disease. Physiother Theory Pract. (2020) 1–10. 10.1080/09593985.2020.1855684 33263260

[49] Physical Activity Policy Health Improvement Directorate. The General Practise Physical Activity Questionnaire (GPPAQ). A Screening Tool to Assess Adult Physical Activity Levels, Within Primary Care. United Kingdom, Physical Activity Policy HID, 18th May, 2009. Contract No.: 11854 (2009).

[50] Schwarzer R Renner B. Health-Specific Self-Efficacy Scales. Freie Universitat Berlin (2009).

[51] Devlin NJ Brooks R. EQ-5D and the EuroQol group: past, present and future. Appl Health Econ Health Policy. (2017) 15:127–37. 10.1007/s40258-017-0310-5 28194657 PMC5343080

[52] Cleemput I Kesteloot K Moons P Vanrenterghem Y Van Hooff JP Squifflet JP. The construct and concurrent validity of the EQ-5D in a renal transplant population. Value Health. (2004) 7:499–509. 10.1111/j.1524-4733.2004.74013.x 15449642

[53] EuroQol Research Foundation. EQ-5D-5L User Guide Online: EuroQol Research Foundation. (2019). Available online at: https://euroqol.org/publications/user-guides/ (accessed February 22, 2021).

[54] van Hout B Janssen MF Feng YS Kohlmann T Busschbach J Golicki D. Interim scoring for the EQ-5D-5L: mapping the EQ-5D-5L to EQ-5D-3L value sets. Value Health. (2012) 15:708–15. 10.1016/j.jval.2012.02.008 22867780

[55] EuroQol Research Foundation. EQ-5D-5L Valuation Crosswalk Index Value Calculator. (2021). Available online at: https://euroqol.org/eq-5d-instruments/eq-5d-5l-about/valuation-standard-value-sets/crosswalk-index-value-calculator/ (accessed June 16, 2021).

[56] Chalder T Berelowitz G Pawlikowska T Watts L Wessely S Wright D. Development of a fatigue scale. J Psychosom Res. (1993) 37:147–53. 10.1016/0022-3999(93)90081-P 8463991

[57] Levey AS Stevens LA Schmid CH Zhang YL Castro AF III Feldman HI. A new equation to estimate glomerular filtration rate. Ann Internal Med. (2009) 150:604–12. 10.7326/0003-4819-150-9-200905050-00006 19414839 PMC2763564

[58] National Kidney Foundation. GFRF Calculator. NKF (2021). Available online at: https://www.kidney.org/professionals/kdoqi/gfr_calculator

[59] Eldridge SM Chan CL Campbell MJ Bond CM Hopewell S Thabane L. CONSORT 2010 statement: extension to randomised pilot and feasibility trials. BMJ. (2016) 355:i5239. 10.1136/bmj.i5239 27777223 PMC5076380

[60] Julious SA. Sample size of 12 per group rule of thumb for a pilot study. Pharm Stat. (2005) 4:287–91. 10.1002/pst.185

[61] Sim J Lewis M. The size of a pilot study for a clinical trial should be calculated in relation to considerations of precision and efficiency. J Clin Epidemiol. (2012) 65:301–8. 10.1016/j.jclinepi.2011.07.011 22169081

[62] Hooper R. Justifying the Sample Size for a Feasibility Study. Research Design Service London. National Institute for Health Research. Available online at: https://www.rds-london.nihr.ac.uk/resources/justify-sample-size-for-a-feasibility-study/ (accessed June 22, 2021).

[63] Patton MQ. Qualitative Research and Evaluation methods. 3rd ed. Thousand Oaks, CA: Sage publications (2002).

[64] Braun V Clarke V. Reflecting on reflexive thematic analysis. Qual Res Sport Exerc Health. (2019) 11:589–97. 10.1080/2159676X.2019.1628806

[65] Malterud K Siersma VD Guassora AD. Sample size in qualitative interview studies: guided by information power. Qual Health Res. (2016) 26:1753–60. 10.1177/1049732315617444 26613970

[66] Braun V Clarke V. To saturate or not to saturate? Questioning data saturation as a useful concept for thematic analysis and sample-size rationales. Qual Res Sport Exerc Health. (2019) 13:1–16. 10.1080/2159676X.2019.1704846

[67] Braun V Clarke V. Using thematic analysis in psychology. Qual Res Psychol. (2006) 3:77–101. 10.1191/1478088706qp063oa

[68] Cherryholmes CH. Notes on pragmatism and scientific realism. Educ Res. (1992) 21:13–7. 10.3102/0013189X021006013

[69] Creswell JW Plano Clark VL. Designing and Conducting Mixed Methods Research. 3rd ed. Los Angeles, CA: Sage (2018).

[70] O'Cathain A Murphy E Nicholl J. Three techniques for integrating data in mixed methods studies. BMJ. (2010) 341:c4587. 10.1136/bmj.c4587 20851841

[71] Heiwe S Jacobson SH. Exercise training for adults with chronic kidney disease. Cochrane Database Syst Rev. (2011) 10:CD003236. 10.1002/14651858.CD003236.pub2 21975737 PMC10183198

[72] Serper M Barankay I Chadha S Shults J Jones LS Olthoff KM. A randomized, controlled, behavioral intervention to promote walking after abdominal organ transplantation: results from the LIFT study. Transpl Int. (2020) 33:632–43. 10.1111/tri.13570 31925833 PMC7239718

[73] Gibson CA Gupta A Greene JL Lee J Mount RR Sullivan DK. Feasibility and acceptability of a televideo physical activity and nutrition program for recent kidney transplant recipients. Pilot Feas Stud. (2020) 6:126. 10.1186/s40814-020-00672-4 32944274 PMC7488333

[74] Eysenbach G. CONSORT-EHEALTH: improving and standardizing evaluation reports of web-based and mobile health interventions. J Med Internet Res. (2011) 13:e126. 10.2196/jmir.1923 22209829 PMC3278112

[75] Schmid-Mohler G Zala P Graf N Witschi P Mueller TF Peter Wuthrich R. Comparison of a behavioral versus an educational weight management intervention after renal transplantation: a randomized controlled trial. Transplant Direct. (2019) 5:e507. 10.1097/TXD.0000000000000936 32095502 PMC7004588

[76] Bradbury K Dennison L Little P Yardley L. Using mixed methods to develop and evaluate an online weight management intervention. Br J Health Psychol. (2015) 20:45–55. 10.1111/bjhp.12125 25406436

[77] Sherrington A Newham JJ Bell R Adamson A McColl E Araujo-Soares V. Systematic review and meta-analysis of internet-delivered interventions providing personalized feedback for weight loss in overweight and obese adults. Obes Rev. (2016) 17:541–51. 10.1111/obr.12396 26948257 PMC4999041

[78] Little P Stuart B Hobbs FR Kelly J Smith ER Bradbury KJ. An internet-based intervention with brief nurse support to manage obesity in primary care (POWeR+): a pragmatic, parallel-group, randomised controlled trial. Lancet Diabetes Endocrinol. (2016) 4:821–8. 10.1016/S2213-8587(16)30099-7 27474214

[79] Williamson DA Bray GA Ryan DH. Is 5% weight loss a satisfactory criterion to define clinically significant weight loss? Obesity. (2015) 23:2319–20. 10.1002/oby.21358 26523739

[80] American American College of Cardiology/American Heart Asssociation Tast Force on Practise Guidelines Obesity Expert Panel. Executive summary: Guidelines 2013 for the management of overweight and obesity in adults: a report of the American College of Cardiology/American Heart Association Task Force on Practice Guidelines and the Obesity Society published by the Obesity Society and American College of Cardiology/American Heart Association Task Force on Practice Guidelines. Based on a systematic review from the The Obesity Expert Panel, 2013. Obesity. (2014) 22(Suppl. 2):S5–39. 10.1002/oby.20821 24961825

[81] Ryan DH Yockey SR. Weight loss and improvement in comorbidity: differences at 5%, 10%, 15%, and over. Curr Obes Rep. (2017) 6:187–94. 10.1007/s13679-017-0262-y 28455679 PMC5497590

[82] Booth S Adams L. The shuttle walking test: a reproducible method for evaluating the impact of shortness of breath on functional capacity in patients with advanced cancer. Thorax. (2001) 56:146. 10.1136/thorax.56.2.146 11209105 PMC1745995

[83] Anwar S Ashraf M Rizvi S Khalid S Delos Santos R Klein C. Impaired 6 minute walk distance predicts poor graft survival in kidney transplant patients.: abstract# D2491. Transplantation. (2014) 98:633. 10.1097/00007890-201407151-02143

[84] Watanabe FT Koch VH Juliani RC Cunha MT. Six-minute walk test in children and adolescents with renal diseases: tolerance, reproducibility and comparison with healthy subjects. Clinics. (2016) 71:22–7. 10.6061/clinics/2016(01)05 26872080 PMC4737086

[85] Wise RA Brown CD. Minimal clinically important differences in the six-minute walk test and the incremental shuttle walking test. Copd. (2005) 2:125–9. 10.1081/COPD-200050527 17136972

[86] Shoemaker MJ Curtis AB Vangsnes E Dickinson MG. Triangulating clinically meaningful change in the six-minute walk test in individuals with chronic heart failure: a systematic review. Cardiopulm Phys Ther J. (2012) 23:5–15. 10.1097/01823246-201223030-00002 22993497 PMC3443464

[87] Shoemaker MJ Curtis AB Vangsnes E Dickinson MG. Clinically meaningful change estimates for the six-minute walk test and daily activity in individuals with chronic heart failure. Cardiopulm Phys Ther J. (2013) 24:21–9. 10.1097/01823246-201324030-00004 23997688 PMC3751711

[88] Bohannon RW Crouch R. Minimal clinically important difference for change in 6-minute walk test distance of adults with pathology: a systematic review. J Eval Clin Pract. (2017) 23:377–81. 10.1111/jep.12629 27592691

[89] Kohl LdM Signori LU Ribeiro RA Silva AMV Moreira PR Dipp T. Prognostic value of the six-minute walk test in end-stage renal disease life expectancy: a prospective cohort study. Clinics. (2012) 67:581–6. 10.6061/clinics/2012(06)06 22760895 PMC3370308

[90] Cesari M Calvani R Canevelli M Aprahamian I de Souto Barreto P Azzolino D. On Schrödinger's cat and evaluation of trials disrupted by the Covid19 pandemic: a critical appraisal. J Frailty Aging. (2021) 10:310–12. 10.14283/jfa.2021.23 34549243 PMC8140750

