# Supplementary material for: The Feasibility and User-Experience of a Digital Health Intervention Designed to Prevent Weight Gain in New Kidney Transplant Recipients—The ExeRTiOn2 Trial
Source: Front Nutr. 2022 May 23;9:887580. doi: 10.3389/fnut.2022.887580 (PMC9168981; doi:10.3389/fnut.2022.887580)
Supplement: Supplementary file 1 [file Data_Sheet_1.docx]

Supplementary Material

# Supplementary Figures and Tables

## S1. Detailed transplant and medical characteristics

| Variable | Total sample | | | DHI group | | | Usual care | | |
| --- | --- | --- | --- | --- | --- | --- | --- | --- | --- |
|  | **Baseline (n=17)** | **3-months (n=15)** | **12-months (n=13)** | **Baseline (n=9)** | **3-months (n=8)** | **12-months**  **(n=6 )** | **Baseline**  **(n=8)** | **3-months**  **(n=7)** | **12-months**  **(n=7)** |
| eGFR epi-CKD (mL/min/1.73m2) | 40 (32 to 60.0) | 43 (40 to 58.0) | 52 (33 to 66) | 42 (29 to 64) | 44 (41.5 to 62.5) | 52.5 (50 to 66) | 40 (33 to 44) | 42 (33 to 50) | 45 (27 to 66) |
| TDD Prednisolone  (Median and IQR) | 5 (5 to 7.5) | 5 (5 to 5) | 5 (5 to 5) | 5 (5 to 5) | 5 (5 to 5) | 5 (5 to 5) | 8.8 (5 to 10) | 5 (5 to 5) | 5 (5 to 5) |
| TDD Tac  (Median and IQR) | 16 (8 to 20) | 12 (5 to 14) | 8 (5 to 10) | 16 (10 to 20) | 13.5 (5.5 to 14) | 8.5 (6 to 10) | 13 (6 to 24) | 6 (4 to 14) | 6 (4 to 14) |
| TDD MMF  (Median and IQR) | 1000 (1000 to 1000) | 1000 (500 to 1000) | 1000 (500 to 1000) | 1000 (500 to 1000) | 1000 (500 to 1000) | 750 (500 to 1000) | 1000 (1000 to 1000) | 1000 (500 to 1000) | 1000 (500 to 1000) |
| Diabetes medical management-insulin only | 3 (17.6%) | 3 (20%) | 3 (23.1%) | 2 (22.2%) | 2 (25%) | 2 (33.3%) | 1 (12.5%) | 1 (14.3%) | 1 (14.3%) |
| Diabetes medical management-oral OR insulin |  | 2 (13.3%) | 3 (23.1%) |  |  |  |  | 2 (28.6%) | 3 (42.9%) |
| Diabetes Diagnosis- Type 1 | 1 (5.9%) | 1 (6.7%) | 1 (7.7%) | 1 (11.1%) | 1 (12.5%) | 1 (16.7%) |  |  |  |
| Diabetes Diagnosis- Type 2 | 2 (11.8%) | 1 (6.7%) | 1 (7.7%) |  |  |  | 2 (25%) | 1 (14.3%) | 1 (14.3%) |
| Diabetes Diagnosis- (PTDM) | 1 (5.4%) | 3 (20%) | 4 (30.8%) | 1 (11.1%) | 1 (12.5%) | 1 (16.7%) |  | 2 (28.6%) | 3 (42.9%) |
| Prescribed antihypertensives | 11 (64.7%) | 11 (73.3%) | 10 (76.9%) | 7 (77.8%) | 6 (75%) | 4 (66.7%) | 4 (50%) | 5 (71.4%) | 6 (85.7%) |
| Number of antihypertensives  (Median, IQR) | 1 (0 to 1) | 1 (0 to 1) | 1 (1 to 1) | 1 (1 to 1) | 1 (0.5 to 1) | 1 (0 to 1) | 0.5 (0 to 1) | 1 (0 to 1) | 1 (1 to 2) |
| SBP (mmHg) | 138.0 (121.0 to 149.0) | 128.0 (125.0 to 146.0) | 130.0 (125.0 to 143.0) | 137.0 (121.0 to 148.0) | 127.5 (121.5 to 134.0) | 133.5 (125.0 to 143.0) | 143.0 (117.5 to 150.0) | 132.0 (126.0 to 156.0) | 130.0 (124.0 to 147.0) |
| DBP (mmHg) | 83.0 (73.0 to 88.0) | 83.0 (73.0 to 90.0) | 83.0 (80.0 to 89.0) | 83.0 (73.0 to 86.0) | 80.5 (71.5 to 92.0) | 84.5 (70.0 to 95.0) | 85.5 (75.0 to 90.5) | 84.0 (77.0 to 89.0) | 83.0 (80.0 to 89.0) |
| RHR (bpm) | 82.0 (74.0 to 88.0) | 78.0 (71.0 to 84.0) | 86.0 (78.0 to 90.0) | 83.0 (74.0 to 88.0) | 79.5 (71.5 to 93.5) | 82.5 (72.0 to 94.0) | 80.0 (71.0 to 90.0) | 77.0 (71.0 to 80.0) | 86.0 (81.0 to 90.0) |
| Episodes of rejection over 12-month trial |  |  | 7 (41.2%) |  |  | 3 (33.3%) |  |  | 4 (50%) |
| Episodes of CMV over 12-month trial  (n, %) |  |  | 10 (58.5%) |  |  | 5 (55.6%) |  |  | 5 (62.5%) |

*Note*. Continuous data presented as median with IQR ranges. Ordinal data is displayed using number of participants (n) and valid proportions (%). eGFR epi-CKD= estimated glomerular filtration rate using the Chronic Kidney Disease Epidemiology Collaboration equation, TDD=total daily dose, Tac= tacrolimus, MMF= mycophenolate mofetil, SBP= systolic blood pressure, DBP= diabetic blood pressure and RHR= resting heart rate. For diabetes management, medical notes were reviewed, and prescribed medications were categorized as insulin only, oral only, insulin and oral. Episodes of CMV were taken from documentation in participants medical notes. Episodes of rejection were categorized by confirmed acute rejection from transplant biopsy reports. PTDM refers to a documented diagnosis of post-transplant diabetes mellitus from medical records

## S2. Secondary outcome data (QUANT)

| Variable | Total sample | | | DHI group | | | Usual care | | |
| --- | --- | --- | --- | --- | --- | --- | --- | --- | --- |
|  | **Baseline (n=17)** | **3-months (n=15)^a^** | **12-months (n=13) ^b^** | **Baseline (n=9)** | **3-months (n=8)^a^** | **12-months**  **(n=6 )** | **Baseline**  **(n=8)** | **3-months**  **(n=7)^a^** | **12-months**  **(n=7 )^b^** |
| Body Weight (kg) | 92.6 (72.0 to 96.1) | 91.7 (69.0 to 103.3) | 93.3 (77.2 to 101.9) | 94.5 (63.0 to 102.0) | 95.0 (66.7 to 105.3) | 94.7 (77.2 to 117.3) | 81.3 (73.6 to 94.6) | 86.2 (75.4 to 96.5) | 93.3 (70.3 to 101.9) |
| BMI (kg/m^2^) | 27.9 (23.9 to 32.9) | 28.9 (25.2 to 33.1) | 29.4 (27.8 to 35.0) | 30.0 (23.9 to 33.6) | 30.6 (23.2 to 34.5) | 32.2 (29.4 to 36.2) | 26.8 (24.6 to 29.8) | 27.1 (25.2 to 32.2) | 28.2 (23.5 to 34.4) |
| Waist circumference (cm) | 108.0 (91.5 to 119.0) | 111.0 (94.5 to 114.0) | 112.0 (99.0 to 120.5) | 108.0 (86.0 to 119.0) | 97.3 (91.3 to 107.0) | 110.5 (95.0 to 120.0) | 105.5 (96.8 to 115.5) | 111.5 (111.0 to 124.0) | 112.0 (111.0 to 129.0) |
| Hip circumference (cm) | 107.2 (98.0 to 110.0) | 109.0 (100.0 to 115.0) | 111.5 (104.5 to 117.5) | 107.5 (107.0 to 110.0) | 105.0 (98.3 to 112.5) | 114.5 (105.0 to 115.0) | 102.3 (95.8 to 113.6) | 109.0 (106.0 to 117.0) | 107.0 (104.0 to 120.0) |
| FTI (kg/m^2^) | 12.9 (10.9 to 20.1) | 15.5 (11.3 to 16.1) | 18.8 (15.7 to 24.5) | 15.5 (12.1 to 20.1) | 15.6 (10.2 to 15.9) | 21.1 (14.8 to 26.4) | 12.1 (10.7 to 18.7) | 12.6 (11.3 to 21.7) | 17.4 (16.6 to 22.5) |
| FM (kg) | 30.1 (22.8 to 44.9) | 33.6 (28.0 to 42.8) | 41.7 (36.2 to 53.6) | 36.2 (24.9 to 47.9) | 33.3 (20.6 to 37.2) | 45.7 (34.4 to 53.7) | 28.3 (22.2 to 44.1) | 39.2 (28.3 to 51.7) | 41.7 (37.9 to 48.8) |
| FM (%) | 38.1 (29.7 to 45.7) | 38.5 (36.1 to 47.1) | 45.6 (39.0 to 51.6) | 38.1 (29.9 to 42.1) | 38.1 (26.8 to 42.1) | 47.3 (37.0 to 55.4) | 37.6 (28.9 to 46.6) | 43.5 (36.6 to 50.6) | 44.6 (41.0 to 47.9) |
| LTI (kg/m^2^) | 12.7 (10.9 to 15.0) | 13.5 (10.9 to 15.8) | 11.8 (10.6 to 13.0) | 12.7 (11.5 to 15.1) | 15.4 (12.2 to 18.9) | 11.8 (9.4 to 13.5) | 12.3 (10.7 to 14.7) | 12.5 (10.9 to 13.5) | 11.8 (11.6 to 12.2) |
| LTM (kg) | 40.5 (30.2 to 44.6) | 39.3 (30.6 to 48.4) | 34.5 (29.3 to 40.4) | 40.7 (29.3 to 46.7) | 48.2 (36.1 to 53.1) | 30.3 (25.8 to 43.8) | 37.2 (32.3 to 42.8) | 32.5 (30.6 to 39.5) | 35.6 (34.3 to 38.8) |
| LTM (%) | 48.2 (35.9 to 57.2) | 48.8 (36.3 to 52.4) | 37.5 (30.2 to 48.4) | 48.2 (39.2 to 57.8) | 50.4 (44.7 to 64.0) | 37.5 (26.7 to 51.4) | 49.0 (35.0 to 55.6) | 40.4 (30.1 to 50.8) | 39.3 (33.7 to 45.3) |
| 6MWD (meters) | 510.0 (450.0 to 540.0) | 515.0 (465.0 to 615.0) | 472.5 (425.0 to 577.5) | 450.0 (450.0 to 540.0) | 525.0 (472.5 to 615.0) | 495.0 (465.0 to 615.0) | 517.5 (435.0 to 570.0) | 507.5 (442.5 to 605.0) | 435.0 (435.0 to 555.0) |
| PWV (m/sec) | 7.4 (6.8 to 9.6) | 7.4 (7.2 to 7.8) | 7.8 (7.1 to 9.9) | 6.9 (6.6 to 7.4) | 6.9 (6.4 to 7.4) | 7.1 (6.2 to 7.9) | 8.6 (7.4 to 9.8) | 7.8 (7.4 to 8.0) | 9.9 (7.6 to 10.8) |
| AI (%) | 23.0 (19.6 to 29.0) | 27.0 (15.8 to 29.5) | 20.3 (18.0 to 26.0) | 22.0 (19.6 to 27.0) | 21.5 (15.8 to 27.0) | 18.7 (18.0 to 19.6) | 28.7 (20.0 to 33.0) | 29.5 (20.5 to 35.5) | 26.0 (21.0 to 27.6) |

*Note.* Median and Interquartile ranges (IQR) are presented for continuous data. Proportion percentages and frequency numbers are shown for categorical data. BMI= body mass index, FTI= fat tissue index, FM= fat mass, LTI= lean tissue index, LTM= lean tissue mass, 6MWD= six-minute walk distance, PWV= pulse wave velocity and AI= Augmentation index. ^a^ indicates for 3-month assessments 7 out of 15 participants completed their assessment virtually due to COVID-19. Therefore, BIA outcomes, waist and hip circumference, six-minute walk test, PWV and AI are reported in a reduced sample (4 in DHI group and 3 in the usual care group). ^b^ indicates at 12-month assessment one participant (UC) declined a face-to-face assessment therefore for face-to-face outcomes the sample will be reduced to 12 out of the 13 remaining participants at 12-month follow-up.

## S3. Figure demonstrating the processes involved when retrospectively mapping the ExeRTiOn DHI to the behaviour change wheel and behaviour change technique taxonomy


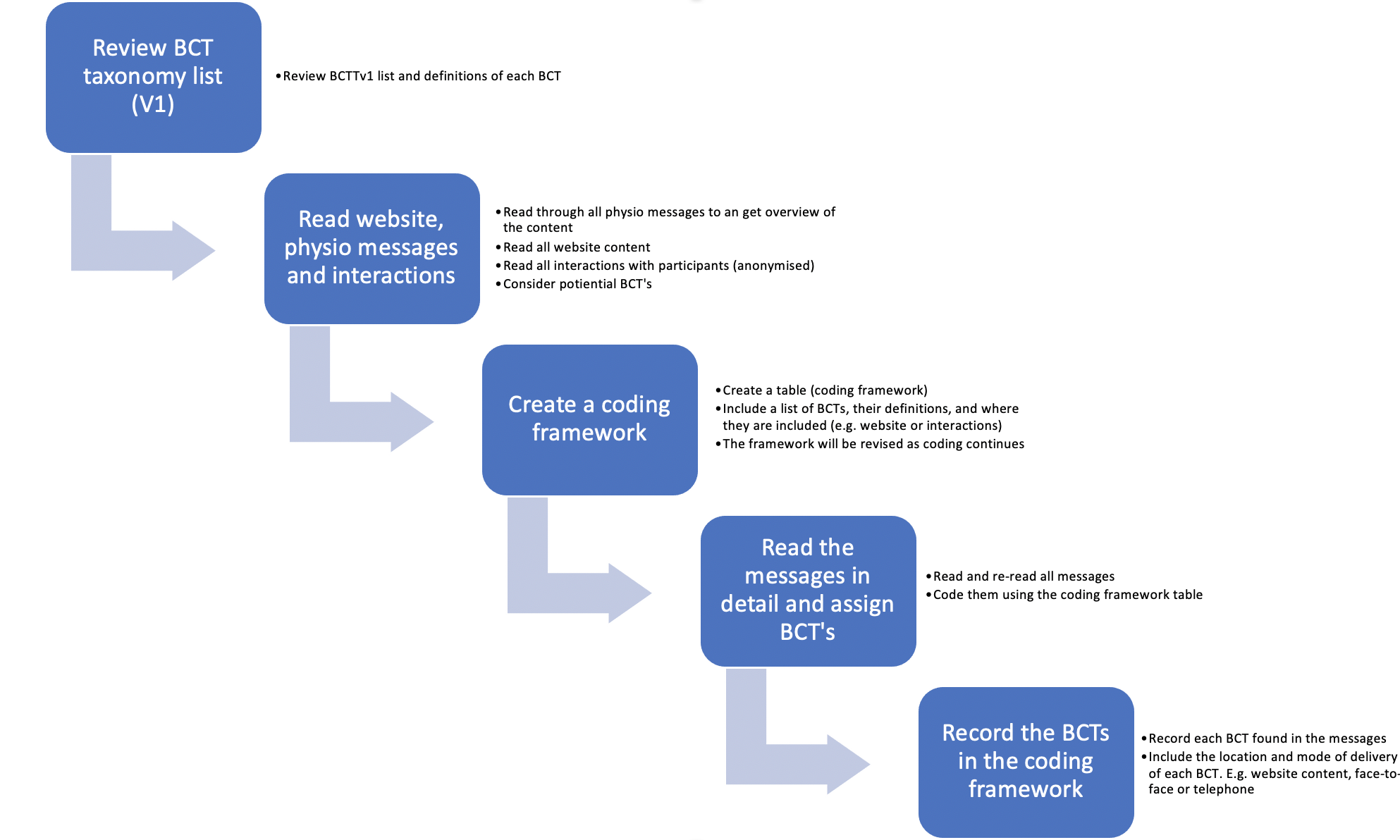


Note. Process involved coding to the behaviour change taxonomy V1 based on Michie et al 2014. BCTTv1= behavior change technique taxonomy version 1(1), BCTs= behavior change techniques

## S4. Questionnaire data

| Variable | Total sample | | | DHI group | | | Usual care group | | |
| --- | --- | --- | --- | --- | --- | --- | --- | --- | --- |
|  | **Baseline (n=17)** | **3-months (n=15)** | **12-months (n=13)** | **Baseline (n=9)** | **3-months (n=8)** | **12-months**  **(n=6 )** | **Baseline**  **(n=8)** | **3-months**  **(n=7)** | **12-months**  **(n=7 )** |
| GPPAQ PAI  -inactive | 14 (82.4%) | 4 (26.7%) | 5 (38.5%) | 7 (77.8%) | 3 (37.5%) | 2 (33.3%) | 7 (87.5%) | 1 (14.3%) | 3 (42.9%) |
| GPPAQ PAI  -mod. inactive |  | 4 (26.7%) | 3 (23.1%) |  | 1 (12.5%) | 1 (16.7%) |  | 3 (42.9%) | 2 (28.6%) |
| GPPAQ PAI  -mod. active | 2 (11.8%) | 4 (26.7%) | 4 (30.8%) | 1 (11.1%) | 3 (37.5%) | 3 (50%) | 1 (12.5%) | 1 (14.3%) | 1 (14.1%) |
| GPPAQ PAI  -active | 1 (5.9%) | 3 (20%) | 1 (7.7%) | 1 (11.1%) | 1 (12.5%) |  |  | 2 (28.6%) | 1 (14.3%) |
| SE-Nutrition | 16 (15 to 20) | 17 (14 to 20) | 19 (14 to 20) | 16 (15 to 20) | 16.5 (14.5 to 19.5) | 16 (12 to 20) | 17.5 (15.0 to 20.0) | 17.0 (14.0 to 20.0) | 20.0 (15.0 to 20.0) |
| SE-Physical Exercise | 15 (13 to 17) | 13 (12 to 18) | 14 (11 to 15) | 13 (11 to 13) | 12 (11 to 15.5) | 13.5 (10 to 15) | 17.5 (15.0 to 19.5) | 17.0 (13.0 to 19.0) | 14.0 (11.0 to 19.0) |
| CFS-Total | 13 (10 to 13) | 10 (7 to 13) | 12 (11 to 16) | 13 (13 to 15) | 12.5 (7.5 to 15.5) | 12 (9 to 16) | 11.5 (8.5 to 13.0) | 9.0 (7.0 to 11.0) | 13.0 (11.0 to 17.0) |
| CFS mental | 4 (3 to 4) | 4 (2 to 4) | 4 (4 to 6) | 4 (3 to 4) | 4 (3 to 6.5) | 5 (4 to 6) | 4.0 (3.0 to 4.0) | 4.0 (1.0 to 4.0) | 4.0 (3.0 to 5.0) |
| CFS Physical | 9 (7 to 11) | 6 (5 to 8) | 8 (6 to 10) | 9 (9 to 11) | 7 (4.5 to 10) | 7 (5 to 10) | 8.0 (5.0 to 9.0) | 6.0 (5.0 to 7.0) | 9.0 (7.0 to 13.0) |
| EQ-5D-index | 0.7 (0.6 to 1.0) | 0.8 (0.7 to 0.9) | 0.8 (0.7 to 0.9) | 0.7 (0.6 to 0.8) | 0.8 (0.7 to 0.9) | 0.7 (0.7 to 0.9) | 0.9 (0.7 to 1.0) | 0.8 (0.7 to 1.0) | 0.8 (0.8 to 1.0) |
| EQ-5D-VAS | 75 (50 to 85) | 80 (65 to 90) | 75 (65 to 85) | 75 (5o to 90) | 85 (70 to 90) | 75 (60 to 85) | 80 (6o to 85) | 80 (65 to 90) | 75 (65 to 92.5) |
| *Note.* GPPAQ PAI= General Practice Physical Activity Questionnaire, PAI=physical activity index, SE=self-efficacy, CFS=chalder fatigue scale, EQ-5D-5L-index and EQ-5D-VAS refers to index values, and visual analogue self-reporting’s on quality of life. Continuous data are summarised using Median (interquartile ranges). Categorical data are summarised using number and proportions. Proportions are for within group sample sizes at each study data collection point | | | | | | | | | |

## S5. Characteristics of the qualitative sub-study sample (n=13)

| Variable |  | Total qualitative interviews (n=13 | DHI Group (n=8) | Usual care Group n=5) |
| --- | --- | --- | --- | --- |
| Age in years | Median (IQR) | 43.0 (33.0 to 59.0) | 36.0 (32.5 to 43.5) | 60 (59 to 60) |
| Males | Number (%) | 7(53.8% ) | 8 (50%) | 3(60%) |
| Ethnicity | White Caucasian | 5 (38.5 %) | 3 (37.5%) | 2 (40%) |
|  | Black African and Caribbean | 6 (46.2%) | 4 (50%) | 2 (40%) |
|  | Asian | 2 (15.4%) | 1 (12.5%) | 1 (20%) |
| Time post-transplant (days) | Median (IQR) | 62.0 (56.0 to 68.0) | 61.0 (57.0 to 69.5) | 65.0 (53.0 to 68.0) |
| RRT before transplant | Number (%) | 19 (76.9%) | 7 (87.5%) | 3 (60%) |
| Number of co-morbidities | One | 7 (53.8%) | 6 (75%) | 1 (20%) |
|  | Two | 5 (38.5%) | 2 (24%) | 3 (60%) |
|  | Three | 1 (7.7%) |  | 1 (20%) |
| Engagement with DHI  (IG only) | Completed all 12 sessions |  | 4 (50%) |  |
|  | Completed tracking only |  | 1 (12.5%) |  |
|  | Completed between 5-10 sessions |  | 3 (37.5%) |  |

*Note.* Due to purposive sampling, medians and interquartile ranges (IQR) are presented for continuous data. Proportion percentages and frequency numbers are shown for categorical data.

Comorbidities included a medical history of diabetes, hypertension, cerebrovascular event, osteoarthritis, brain hemorrhage, cardiovascular disease, cancer or respiratory disease and RRT=renal replacement therapy

## S6. Topic guide example

**
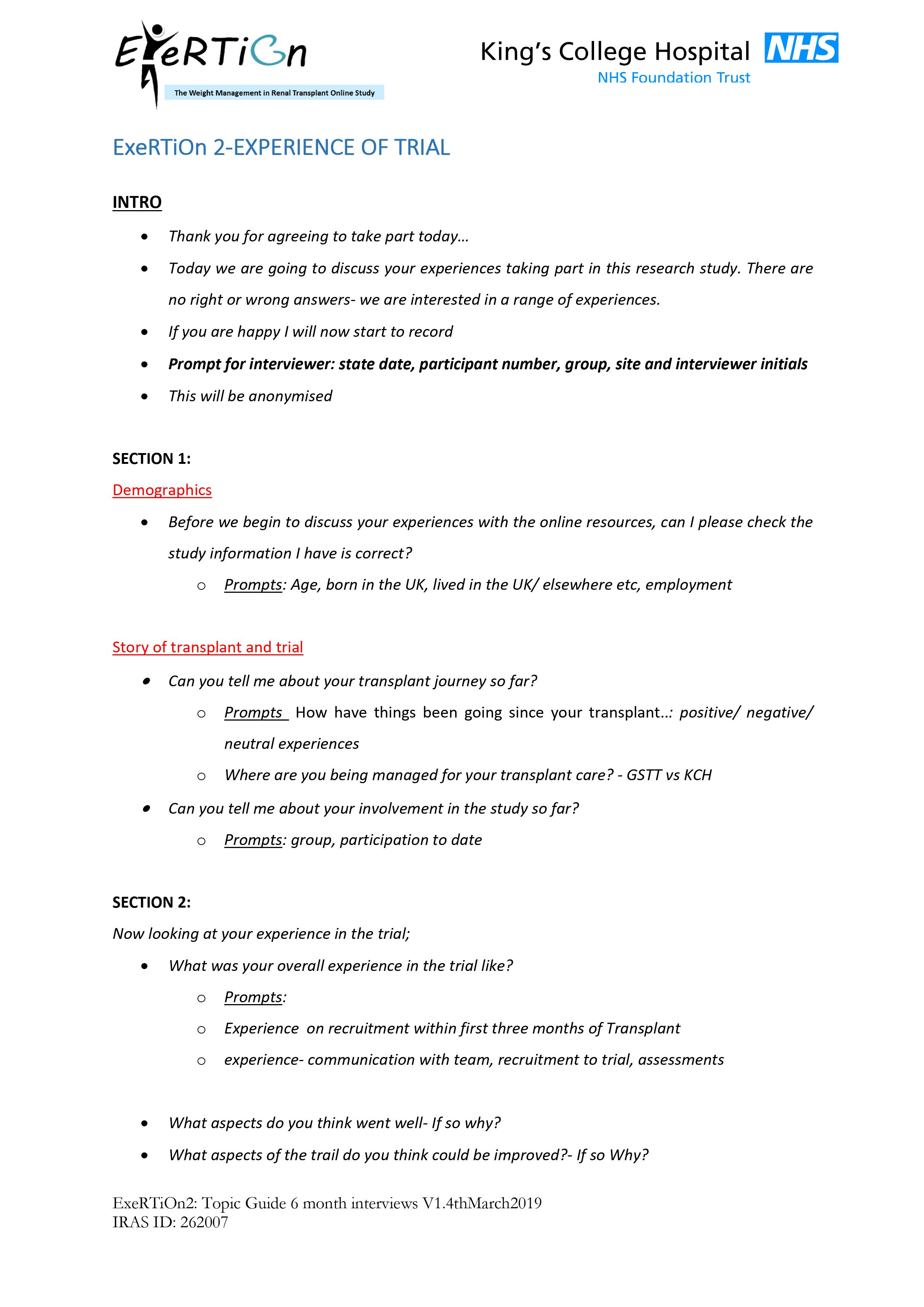
**

**
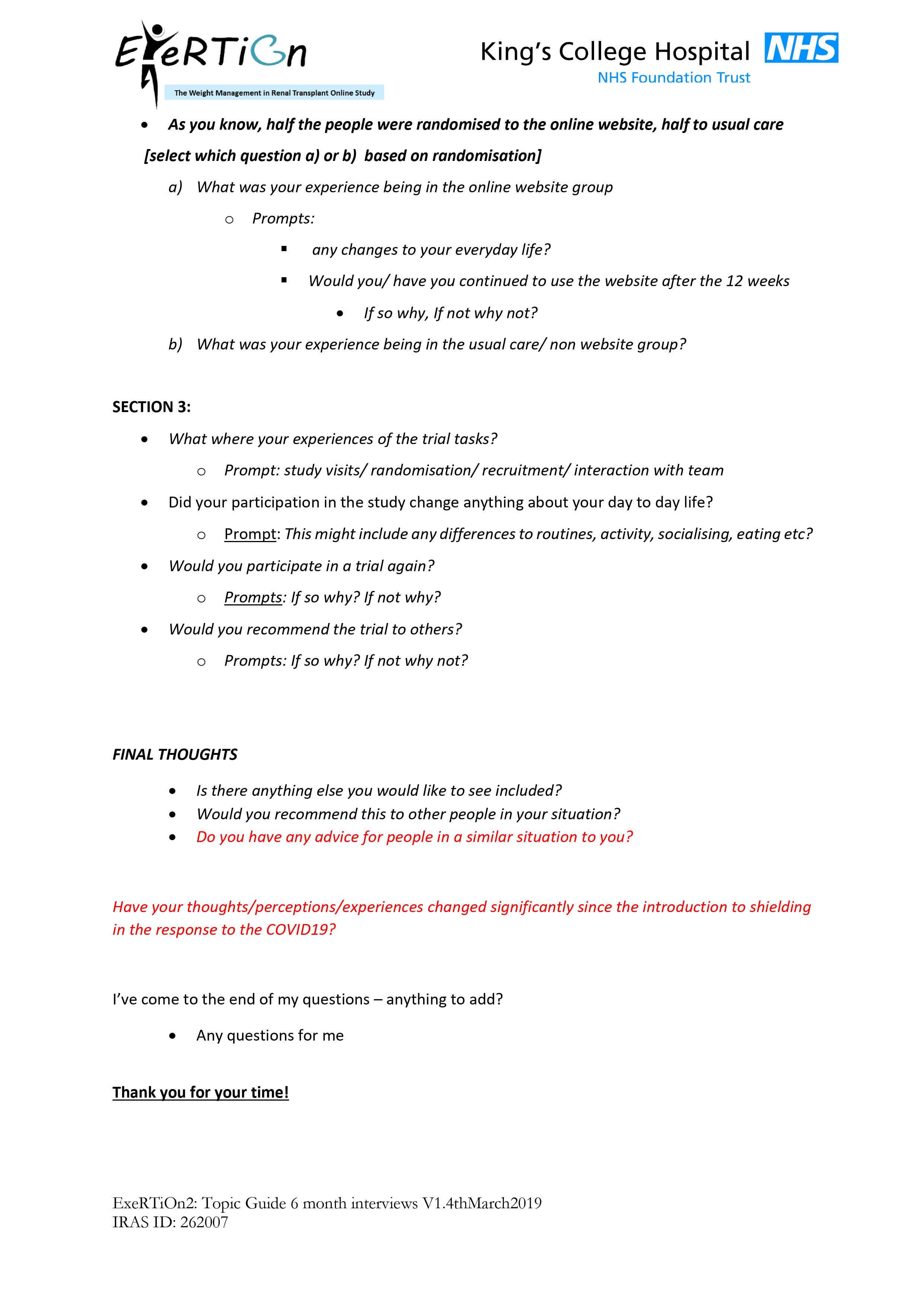
**

References

1. Michie S, Richardson M, Johnston M, Abraham C, Francis J, Hardeman W, et al. The behavior change technique taxonomy (v1) of 93 hierarchically clustered techniques: building an international consensus for the reporting of behavior change interventions. Ann Behav Med. 2013;46(1):81-95.
